# Relationship of mechanical impact magnitude to neurologic dysfunction severity in a rat traumatic brain injury model

**DOI:** 10.1371/journal.pone.0178186

**Published:** 2017-05-26

**Authors:** Tsung-Hsun Hsieh, Jing-Wei Kang, Jing-Huei Lai, Ying-Zu Huang, Alexander Rotenberg, Kai-Yun Chen, Jia-Yi Wang, Shu-Yen Chan, Shih-Ching Chen, Yung-Hsiao Chiang, Chih-Wei Peng

**Affiliations:** 1 Department of Physical Therapy and Graduate Institute of Rehabilitation Science, College of Medicine, Chang Gung University, Taoyuan, Taiwan; 2 Neuroscience Research Center, Chang Gung Memorial Hospital, Linkou, Taiwan; 3 Graduate Institute of Neural Regenerative Medicine, Taipei Medical University, Taipei, Taiwan; 4 Center for Neurotrauma and Neuroregeneration, Taipei Medical University, Taipei, Taiwan; 5 School of Medicine, Taipei Medical University, Taipei, Taiwan; 6 Neuroscience Research Center and Department of Neurology, Chang Gung Memorial Hospital, Taoyuan, Taiwan; 7 Department of Neurology, Boston Children’s Hospital, Harvard Medical School, Boston, Massachusetts, United States of America; 8 Graduate Institute of Medical Sciences, College of Medicine, Taipei Medical University, Taipei, Taiwan; 9 Department of Physical Medicine and Rehabilitation, Taipei Medical University Hospital, Taipei, Taiwan; 10 Department of Physical Medicine and Rehabilitation, School of Medicine, College of Medicine, Taipei Medical University, Taipei, Taiwan; 11 Department of Neurosurgery, Taipei Medical University Hospital, Taipei, Taiwan; 12 School of Biomedical Engineering, College of Biomedical Engineering, Taipei Medical University, Taipei, Taiwan; 13 International Ph.D. Program in Biomedical Engineering, College of Biomedical Engineering, Taipei Medical University, Taipei, Taiwan; University of South Florida, UNITED STATES

## Abstract

**Objective:**

Traumatic brain injury (TBI) is a major brain injury type commonly caused by traffic accidents, falls, violence, or sports injuries. To obtain mechanistic insights about TBI, experimental animal models such as weight-drop-induced TBI in rats have been developed to mimic closed-head injury in humans. However, the relationship between the mechanical impact level and neurological severity following weight-drop-induced TBI remains uncertain. In this study, we comprehensively investigated the relationship between physical impact and graded severity at various weight-drop heights.

**Approach:**

The acceleration, impact force, and displacement during the impact were accurately measured using an accelerometer, a pressure sensor, and a high-speed camera, respectively. In addition, the longitudinal changes in neurological deficits and balance function were investigated at 1, 4, and 7 days post TBI lesion. The inflammatory expression markers tested by Western blot analysis, including glial fibrillary acidic protein, beta-amyloid precursor protein, and bone marrow tyrosine kinase gene in chromosome X, in the frontal cortex, hippocampus, and corpus callosum were investigated at 1 and 7 days post-lesion.

**Results:**

Gradations in impact pressure produced progressive degrees of injury severity in the neurological score and balance function. Western blot analysis demonstrated that all inflammatory expression markers were increased at 1 and 7 days post-impact injury when compared to the sham control rats. The severity of neurologic dysfunction and induction in inflammatory markers strongly correlated with the graded mechanical impact levels.

**Conclusions:**

We conclude that the weight-drop-induced TBI model can produce graded brain injury and induction of neurobehavioral deficits and may have translational relevance to developing therapeutic strategies for TBI.

## Introduction

Traumatic brain injury (TBI) is one of the most common brain injuries caused by an external mechanical force, such as crushing, rapid acceleration or deceleration impact, and projectile penetration [1]. TBI is estimated to affect approximately 1.7 million residents, accounting for an expenditure of more than $76.5 billion in medical care systems every year in the United States [2]. Following TBI, temporary or permanent impairment of cognitive, physical, and psychosocial functions develops depending on the severity of injury [3]. To provide a more stable and controllable environment and obtain detailed mechanical insights into TBI, several animal models of TBI have been developed. One type of rodent model, known as weight-drop models, such as Marmarou’s impact acceleration model, has been widely used to mimic diffuse axonal injury and concussion caused by falls or motor vehicle accidents in individuals with TBI [4, 5]. Furthermore, in this model, Marmarou’s weight-drop procedures can provide an easy and inexpensive method for producing graded brain injury in animals by simply altering the height from which the weights are dropped (1–2 m) [4, 5]. However, a high mortality rate and low reproducibility after severe injury are the two main disadvantages of this model [4–6].

Previous studies have demonstrated that TBI induces sensorimotor and cognitive impairments in weight-drop-induced TBI models [7, 8]. Although the detailed pathophysiology and TBI markers following TBI are currently under investigation, recent research shows significant post-TBI increases in glial fibrillary acidic protein (GFAP), bone marrow tyrosine kinase gene in chromosome X (BMX), and beta-amyloid precursor protein (APP), which may represent the levels of inflammatory or axonal injury and thus act as indicators of trauma severity [9–16].

For translational purposes, identifying and comparing the mechanical or kinematic properties during impact and the induced brain injury level between animals and humans is crucial. Previous studies have demonstrated that the level of mechanical responses such as linear acceleration or velocity during impact is associated with traumatic axonal injury or severity of behavior at 24 h post-injury [17, 18]. However, detailed and accurate data regarding the external mechanical impact force, rapid acceleration, or deceleration that result in brain damage remain insufficient. Furthermore, although weight-drop-induced TBI in rats has been employed in various neuroscience studies [6, 19, 20], the literature regarding the time-course changes in neurobehavioral function and pathophysiological processes following TBI, which may provide insights into the underlying pathophysiology of the disease for future diagnostic purposes and therapeutic applications, is scant. Furthermore, the physiological and behavioral responses to various impacts to the brain similar to injury mechanisms in humans have rarely been investigated in animal models. The detailed relationship between the impact force and the severity of brain damage has not yet been fully explored. This lack of information regarding TBI animals may restrict the use of the knowledge obtained from TBI animal models. The present study applied a weight-drop TBI model designed previously [4, 5] and used an accelerometer, a pressure sensor, and a high-speed camera to accurately measure the acceleration, impact force, and displacement, respectively. Furthermore, the measured data were used to investigate the relationship between the mechanical impact level and the severity of neurological and motor behavioral changes in rats.

## Methods

Fifty-two male Sprague—Dawley rats (BioLASCO Taiwan Co., Ltd, Yilan, Taiwan) weighing between 364 and 425 g (i.e., 10–12 weeks of age) were used for the present experiment. All experimental procedures were preapproved by the Institutional Animal Care and Use Committee (IACUC) of Taipei Medical University (TMU) and followed by the TMU IACUC guidelines to treat animals humanely and reduce animal suffering by use of appropriate anesthesia and analgesics (IACUC Approval No. LAC-2013-0199). Rats were housed on a 12-h light/dark cycle in a temperature- and humidity-controlled animal center until experimental use at TMU. Animals were monitored twice daily prior and post-lesion and animal were humanely euthanized according to accepted pre-established endpoint criteria: loss of >20% body weight, labored breathing, hunched body, and lethargic animal. Furthermore, at the end of the study, all animals were humanely euthanized via carbon dioxide inhalation followed by cervical dislocation.

### Induction of brain injury and monitoring of impact parameters

To minimize animal suffering and distress during surgery, animals were anesthetized using intraperitoneal injection of tiletamine—zolazepam (50 mg/kg, i.p.; Zoletil, Vibac, France) and xylazine (10 mg/kg; Rompun, Bayer, Leverkusen, Germany) 30 min prior to impact. To record the mechanical impact of the head during weight-drop, pressure was recorded using a load cell sensor (10 mm in diameter, 3 mm in thickness; Interface LBS-250, Scottsdale, AZ, USA) fixed to the central portion of the rat skull vault between the bregma and lambdoid sutures. To induce TBI, Marmarou’s impact acceleration model was modified and applied; rats were placed prone on flexible foam and were secured in place by using two elastic belts. A Plexiglas tube was then positioned vertically, and the lower end of the tube was centered directly above the pressure sensor. TBI was induced using a 450-g brass weight falling from 1, 1.5, and 2 m through a vertical transparent Plexiglas tube. The impact response was recorded by the load cell fixed on the rat skull. All the signals were measured using the data acquisition system (Biopac MP 36, Santa Barbara, CA, USA). Sham control TBI rats underwent the same surgical procedures, but did not receive weight-drop induced TBI. Body temperature was monitored with a rectal probe throughout surgery, and the temperature was maintained at 37.0 ± 0.5°C using an adjustable heating pad during recovery from anesthesia.

**Fig 1:**
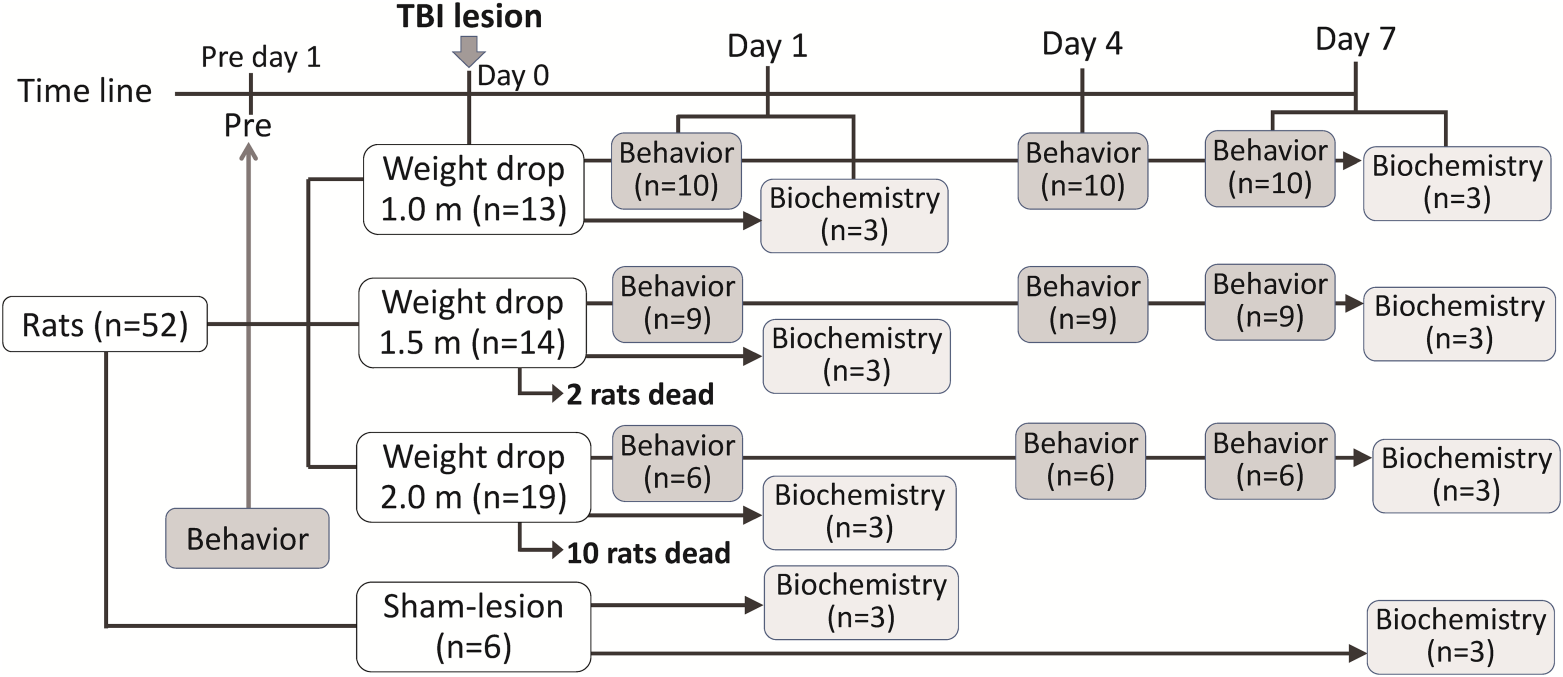
Instrumentation setup of the modified weight-drop-induced head injury model. Kinematic information during impact was captured using a high-speed camera at a rate of 1200 frames/s. The impact force was measured using the miniature load cell that was fixed to the central portion of the skull vault of the rat. The linear acceleration response of the rat head was recorded using a modified accelerometer.

To record the kinematic changes in the head during impact, the impact event was captured using a high-speed video camera (EX-F1, Casio, Tokyo, Japan) (Fig 1). The high-speed video was recorded at 1200 frames/s during each experiment. Furthermore, the linear response of acceleration or deceleration during impact on the rat head was measured using an accelerometer (Model 3225M37, Dytran Instrument Inc., CA, USA) attached on the lateral side of the rat face. Pressure and acceleration signals were digitized and recorded at a sampling rate of 10 kHz by using the Biopac data acquisition system (MP36, BIOPAC System, Goleta, CA, USA).

To analyze the changes in displacement, velocity, and acceleration during impact, a high-speed digital camera was used to record the displacement of the rat head during impact. Black and white line markers were painted on the impactor and helmet for subsequent image tracking analysis through video recordings (see S1 Video for demonstration). The changes in displacement *(x)* of the rat head during impact were determined by tracking the attached impactor and helmeted head by using an image analysis program written in MATLAB. The relationship among displacement *(x)*, velocity *(v)*, and acceleration *(a)* of the rat head with time *(t)* during impact can be formulated as follows:

x=displacement (1)

$$v=\frac{dx}{dt}$$ (2)

$$a=\frac{dv}{dt}$$ (3)

Change in velocity (*v*) of the rat head was calculated by differentiating displacement-time histories of the head from the recorded video data. In addition, the acceleration-time history was calculated using velocity-time histories. Moreover, the acceleration calculated using digital image analysis was compared with that measured by the accelerometer. Thus, peak instantaneous acceleration and deceleration of the rat head were determined using the acceleration-time curve recorded using the digital camera and accelerometer. After surgery, animals were daily monitored for pain and distress. Animals were administered Ketorolac analgesics for 48 h after surgery if pain and distress behaviors were observed.

### Behavioral assessments

#### Neurological function

The modified neurological severity score (mNSS) is a multifunctional evaluation scale that comprises motor, sensory, reflex, and balance tests. The rats receive one point when they are unable to perform the test or lose the tested reflex. Therefore, the score is proportional to the injury severity. The test scale ranges from 0 to 18 (normal score, 0; maximal deficit score, 18).

#### Beam walking test

The beam walking test was used to evaluate fine motor coordination and balance function [21]. Rats were trained to walk from one end to the opposite end of a narrow plastic beam (80 cm long, 1.5 cm wide) at least five times before formal recording. In each test trial, the animal performance was videotaped, and the average elapsed time to traverse the beam in five trials was then calculated [11].

### Western blot

We extracted brain tissue samples from the rats 1 day and 7 days post TBI to quantify the injury severity. The rats were rapidly anesthetized with sodium pentobarbitone (60 mg/kg i.p., Sweden) and then decapitated. Samples were extracted from the frontal cortex (FC), corpus callosum (CC), and hippocampus (H). The samples were then lysed with radioimmunoprecipitation assay buffer. For Western blot analysis, protein cell lysates (30 μg) were resolved on 10% sodium dodecyl sulfate polyacrylamide gel electrophoresis gel and transferred to nitrocellulose membranes. Finally, the membranes with blots were incubated overnight with primary antibodies (anti-actin, 1:2000, Chemicon; anti-GFAP, 1:1000, Chemicon; anti-BMX, 1:1000, BD Biosciences; anti-APP, 1:1000, Novus) at 4°C. The densities of the protein bands were digitized, and the ratio of the ERT density to the beta-actin or alpha-tubulin density was compared among the groups.

### Experimental design

**Fig 2:**
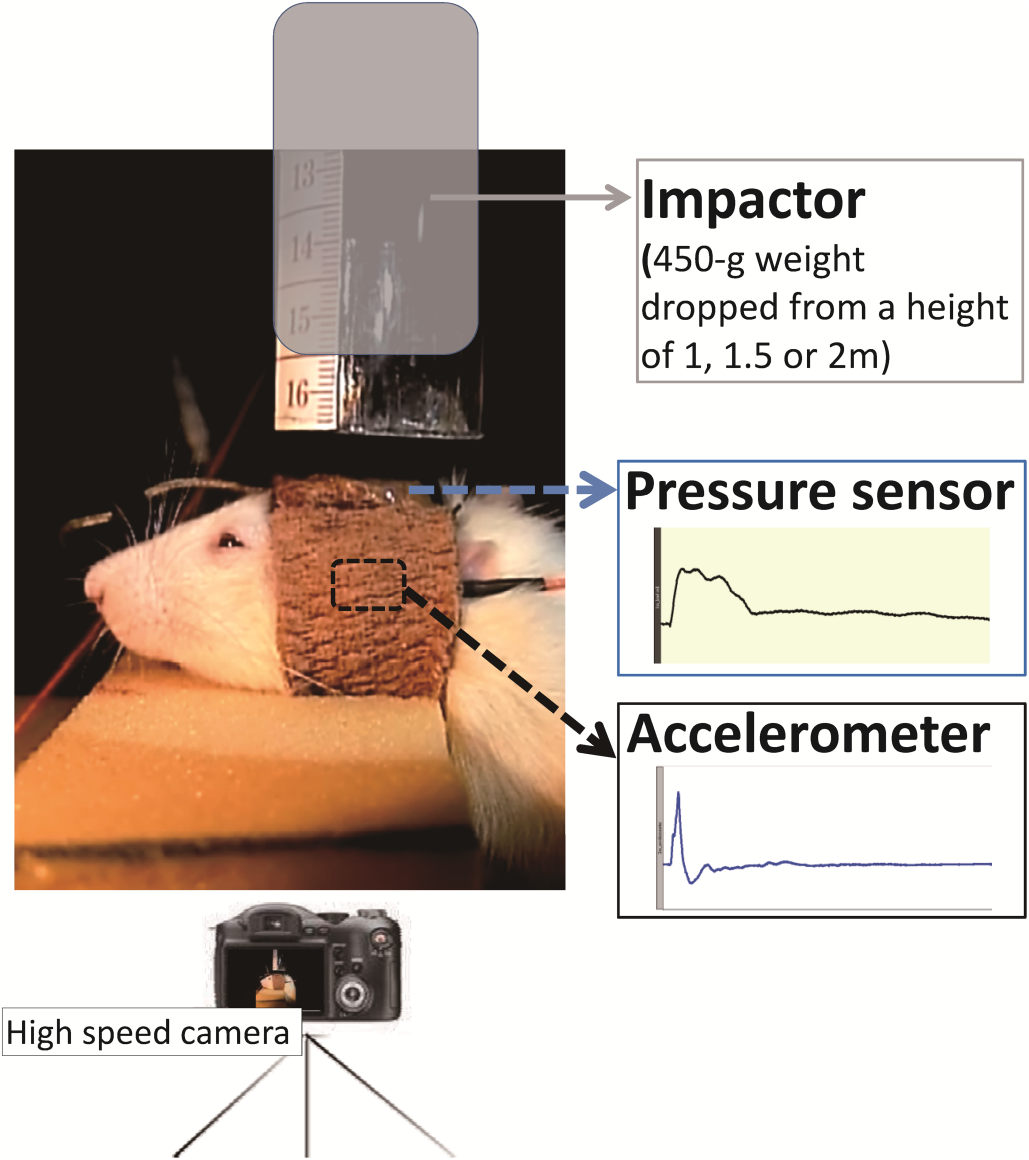
Study design of time-course analysis of behavioral and biochemical recordings following TBI lesion. Behavioral tests including mNSS and beam walking tests were performed before lesion and on days 1, 4, and 7 post-lesion. Western blotting tests were performed on days 1 and 7 post-lesion to quantify the injury severity following weight-drop-induced head injury.

The rats were divided into four groups. A well-trained examiner, blinded for the type of injury, performed all examinations before and after injury. Behavioral alterations were assessed before lesion and on days 1, 4, and 7 post-lesion by using beam walking and mNSS tests. The animals with weight-drop induced were trained and pre-tested for these tasks at least 3 days before TBI lesion to establish baseline data. After training and habituation, all behavioral test sessions were performed at our set time points under the same environmental conditions. After behavioral tests on days 1 and 7 post-lesion, part of rats (*n* = 3 at each timepoint in each group) were sacrificed for western blot analysis (Fig 2).

### Statistical analysis

For statistical analysis of all behavioral measurements, a two-way repeated measure analysis of variance (ANOVARM) with group (1-, 1.5-, and 2-m weight-drop groups) and time factors (i.e., before lesion and 1, 4, and 7 days post-lesion) was performed. Multiple within-subject comparisons were performed using post hoc least significant difference (LSD) analysis when the main effect of time was significant. Furthermore, we used linear Regression test to determine the coefficient of determination (R^2^) between the mechanical impact level (i.e., impact force, acceleration and impact height), the severity of brain injury and neurobehavioral function. Biochemistry comparisons between rats in the sham-lesion group and various fall height groups were performed by one-way ANOVA with LSD multiple comparison post hoc test. Data were analyzed using SPSS version 17.0 (SPSS Inc., USA) with the significance level set at *p* < 0.05 for each assessment. All data are presented as the average standard error of the mean (SEM).

## Results

### Mortality rates

Various mortality rates were observed among the groups depending on the fall height. A 50% (10/20) mortality rate was observed following weight-drop induced TBI from 2 m. In the 1.5-m fall height group, a mortality rate of 12.5% (2/16) was observed. No animal deaths occurred following injury in the 1-m fall height group. All of the deaths of experimental animals occur immediately (< 30 min) followed by the induction of brain injury.

### Biomechanical responses

**Fig 3:**
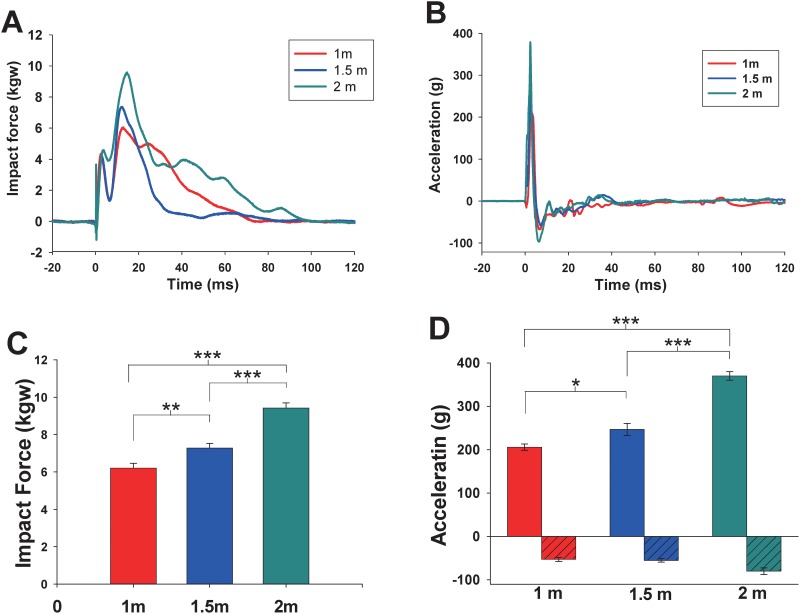
Representative trace of impact force (a) and acceleration (b) during impact from 1-, 1.5-, and 2-m drop tests. Average peak impact force (c) and acceleration (d) from 1-, 1.5-, and 2-m drop tests. The positive bars represent peak acceleration response at the first impact, and negative bars represent rebound deceleration. *p < 0.05; **p < 0.01; ***p < 0.001 per group pair comparison by using the post hoc LSD test. Data are expressed as the mean ± SEM.

The response of impact force and acceleration waveforms of 1, 1.5, and 2 m are shown in Fig 3A and 3B. The averaged maximal impact forces during the 1-, 1.5-, and 2-m drops were 6.21 ± 0.25, 7.28 ± 0.25, and 9.43 ± 0.27 kgw, respectively. During the impact events, the average peak head accelerations recorded by the accelerometer for the 1-, 1.5-, and 2-m groups were 205.96 ± 7.34, 246.83 ± 13.96, and 370.28 ± 9.98 g, respectively (Fig 3C). The rebound head accelerations for the 1-, 1.5-, and 2-m groups were 52.82 ± 4.69, 55.03 ± 4.14, and 80.04 ± 7.68 g, respectively (Fig 3D) (S1 Data).

**Fig 4:**
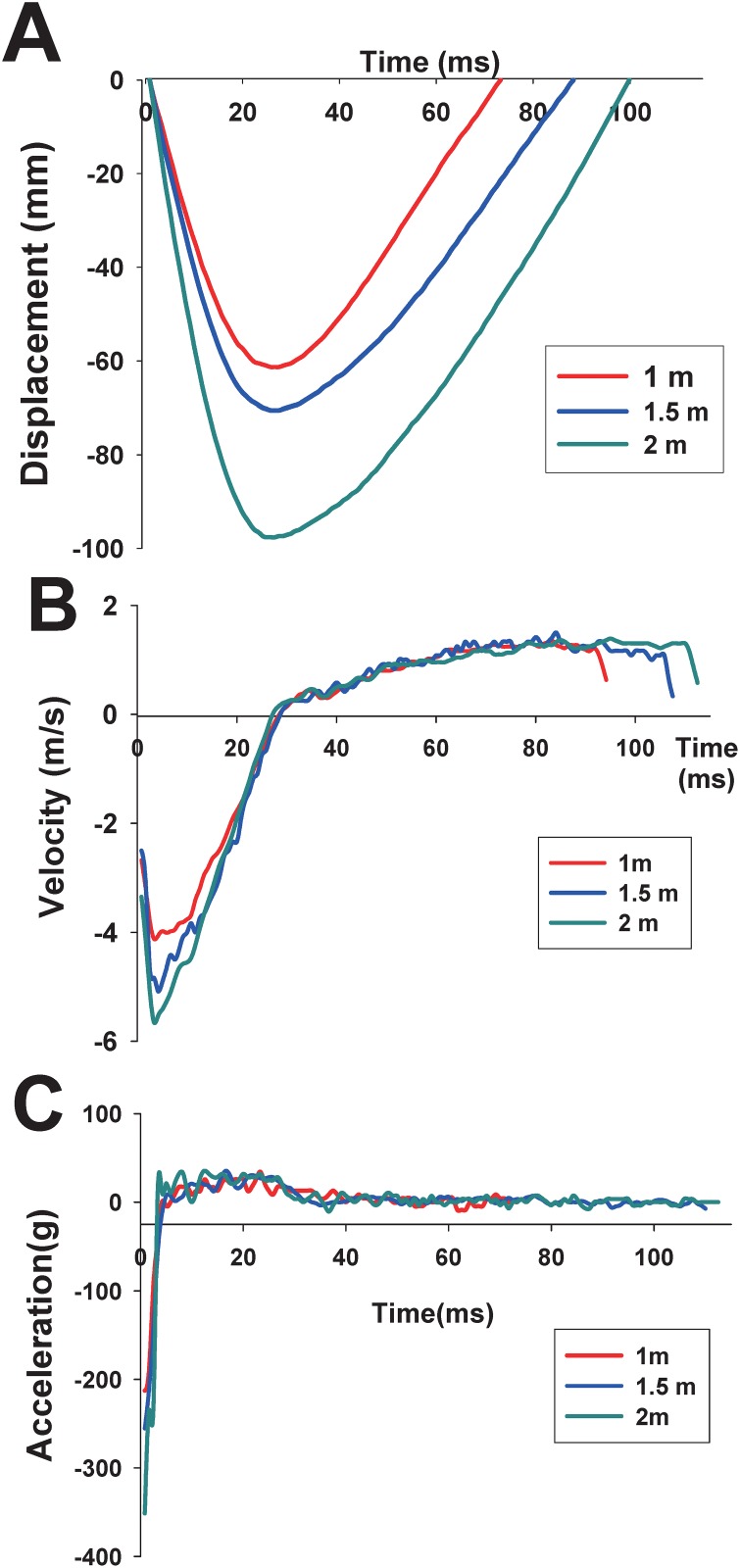
Representative time history curves of the rat head acceleration recorded using an accelerometer from (a) 1-m, (b) 1.5-m, and (c) 2-m drop tests. (a) The representative time-history curve of displacement was captured using a high-speed video camera. (b) The representative time-history curve of velocity was obtained by differentiating the time-history curves of displacement. (c) The representative time-history curve of acceleration was obtained by differentiating the time-history curves of velocity.

The impact displacement trace was captured by the high-speed video camera and is presented in Fig 4A. The changes in velocity and acceleration of the rat head were determined by differentiating the linear head displacement-time histories (Fig 4B and 4C). The average peak displacements, velocities, and accelerations for the 1-, 1.5-, and 2-m groups are summarized in Table 1.

**Table 1:** Biomechanical results of the 450-g brass weight-dropping from 1, 1.5, and 2 m.

Drop height	Peak displacement (mm)	Peak velocity (m/s)	Peak acceleration (g)
**1 m**	62.78 ± 3.50	4.15 ± 0.50	211.19 ± 11.12
**1.5 m**	75.81 ± 6.41	5.05 ± 0.38	249.24 ± 5.82
**2 m**	87.12 ± 7.31	5.94 ± 0.53	323.83 ± 16.82

**Fig 5:**
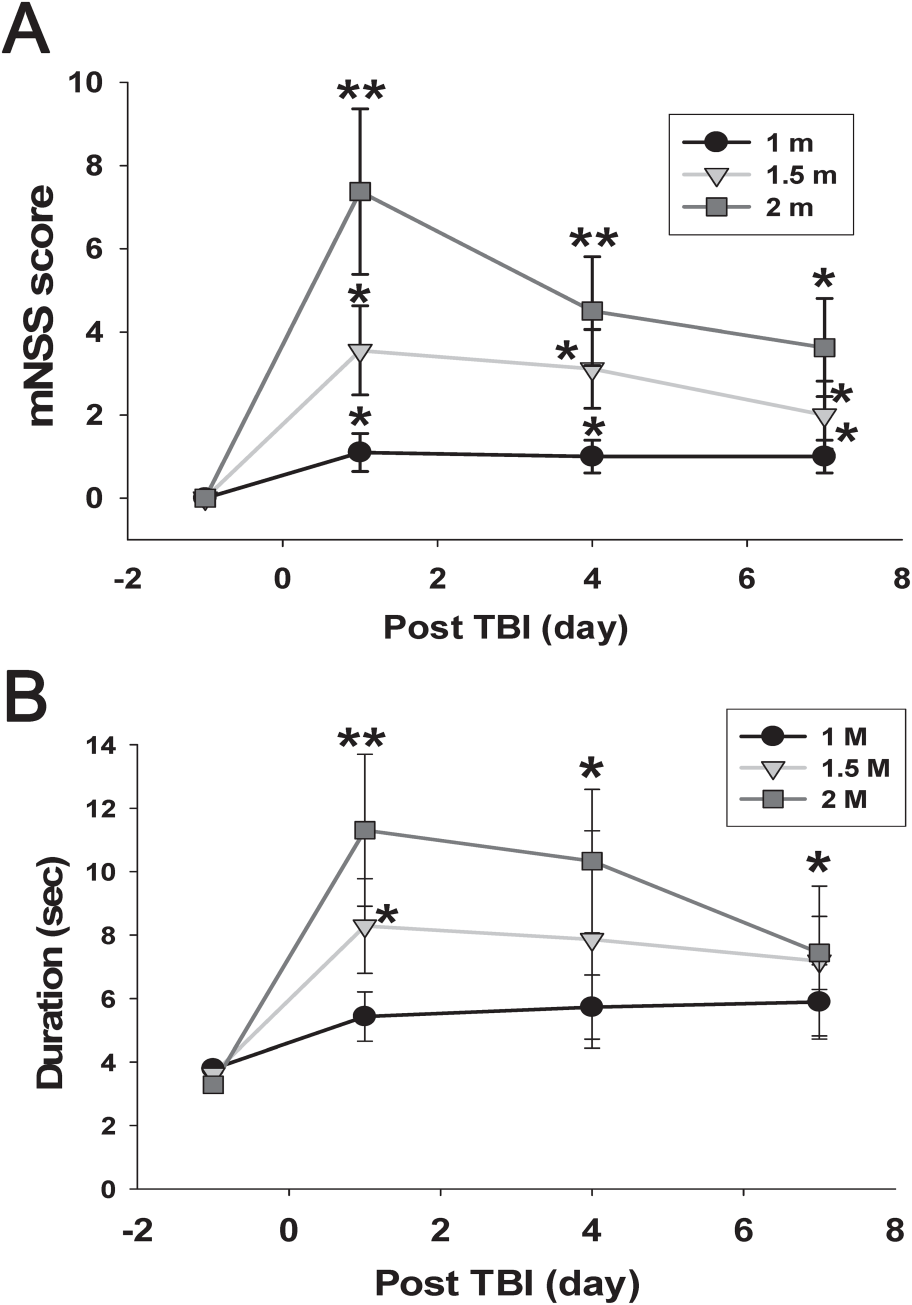
Neurological function evaluated using the mNSS to assess the initial disability before brain injury and on days 1, 4, and 7 following weight-drop-induced brain injury at various fall heights (A). The mNSS in 1, 1.5 and 2 m weight-drop group were persistently higher than that of pre-lesion baseline value after TBI over the complete 7-day observation period. Motor balance function was assessed using the beam walking test (B). Pre- and post-TBI mean ± SEM walking ability as measured by time (s) to traverse an elevated beam for various fall heights. The 2-m weight-drop group exhibited significant impairment on day 1 and remained consistent up to day 7 after the weight-drop lesion when compared with the pre-lesion baseline value. *p < 0.05, **p < 0.01 as compared to pre-operative values (*n* = 10 in 1-m weight-drop group, *n* = 9 in 1.5-m weight-drop group, *n* = 6 in 2-m weight-drop group).

For behavioral tests, neurological and balance functions were assessed 1 day prior to the weight-drop treatment and 1, 4, and 7 days after the weight-drop treatment. Group results for the three fall height groups are presented in Fig 5A and 5B. Fig 5A illustrates the pre- and post-TBI time-course changes in the mNSS. A two-factor ANOVARM on the mNSS over the 7 days showed a significant time × group interaction (F_6,72_ = 4.60, *p* = 0.001) as well as significant time (F_3,72_ = 22.93, *p* < 0.001) and group (F_2,24_ = 5.15, *p* = 0.014) effects. Post hoc LSD analysis revealed that the mNSS in the 1-m, 1.5m and 2-m group was significantly increased on day 1 and remained consistent up to day 7 after the weight-drop experiment compared with the pre-lesion level.

Regarding the beam walking test, a two-factor ANOVARM revealed significant main effects of time (F_3,66_ = 9.218, *p* < 0.0001) but no significant time × group interaction (F_6,66_ = 1.31, *p* = 0.26). Compared with the pre-lesion level, the beam walking test in 2-m group showed a statistically significant increase on day 1 and remained consistent up to day 7 after the weight-drop lesion, but the 1.5 m group did not reach statistical significance up to day 4 post-lesion. In the 1-m group, no significant differences were found in beam balance test when compared to pre-surgery baseline data (all p > 0.05) (Fig 5B).

**Fig 6:**
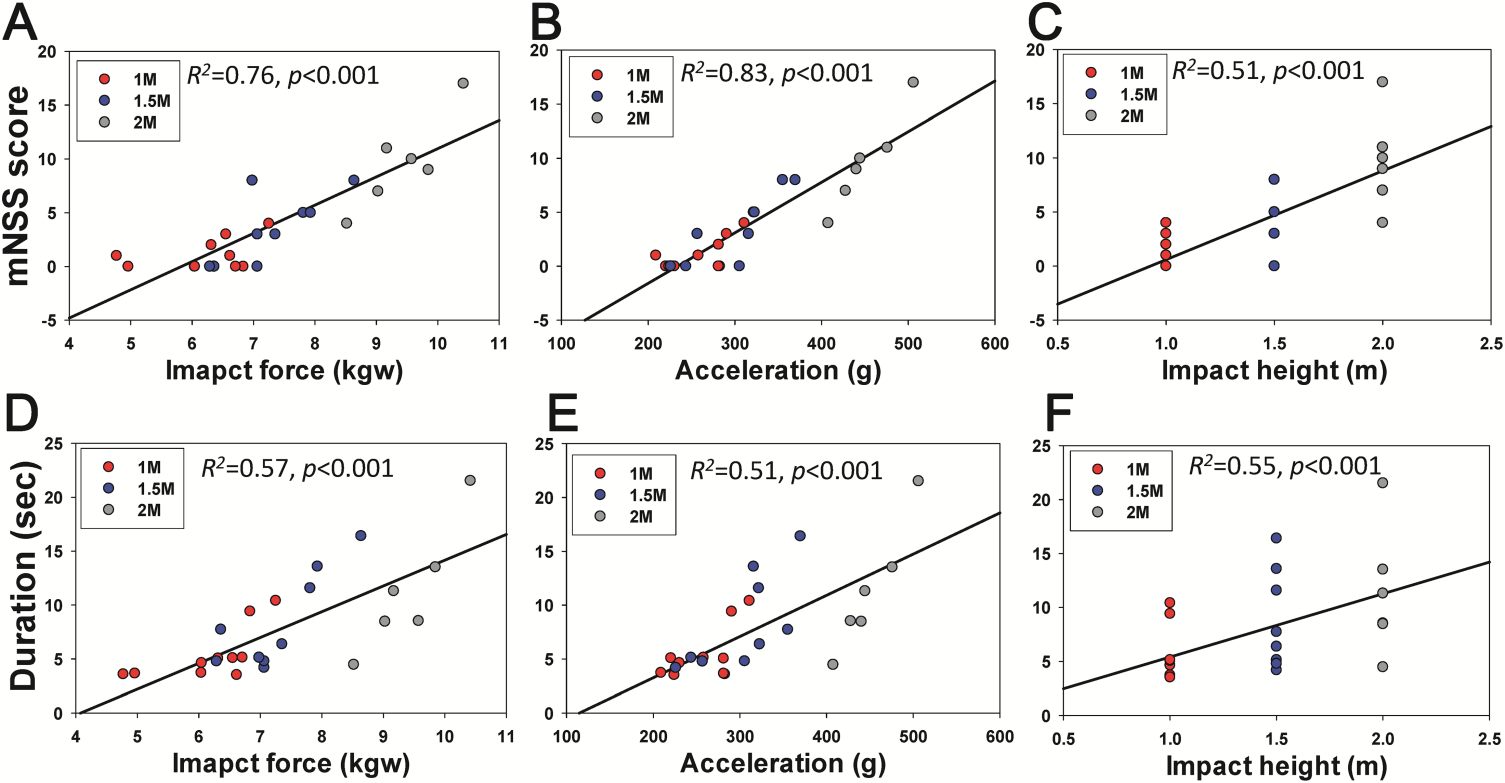
The correlation between the neurobehavioral deficits at day 1 post-lesion and the impact force, acceleration and impact height during weight-drop. There was a significant positive correlation between the mNSS score and the level of impact force (A), acceleration (B) and impact height (C) during weight-drop. The beam balance impairment was also found to have significant positive correlation in the level of impact force (D), acceleration (E) and impact height (F) during weight-drop.

To understand the relationship between physical impact and graded severity at various weight-drop heights, we then measured the neurological and balance functions on day 1 post-injury to correlate with the impact force, peak to peak acceleration and impact height during weight-drop. The linear repression analysis showed a significant positive correlation between the mNSS score and impact force (R^2^ = 0.76, *p* <0.001), acceleration (R^2^ = 0.83, *p* <0.001) and impact height (R^2^ = 0.51, *p* <0.001) (Fig 6A–6C). We also observed a significant positive correlation between the severity of balance function and the impact force (R^2^ = 0.57, *p* <0.001), acceleration (R^2^ = 0.51, *p* <0.001) and impact height (R^2^ = 0.55, *p* <0.001) (Fig 6D–6F).

**Fig 7:**
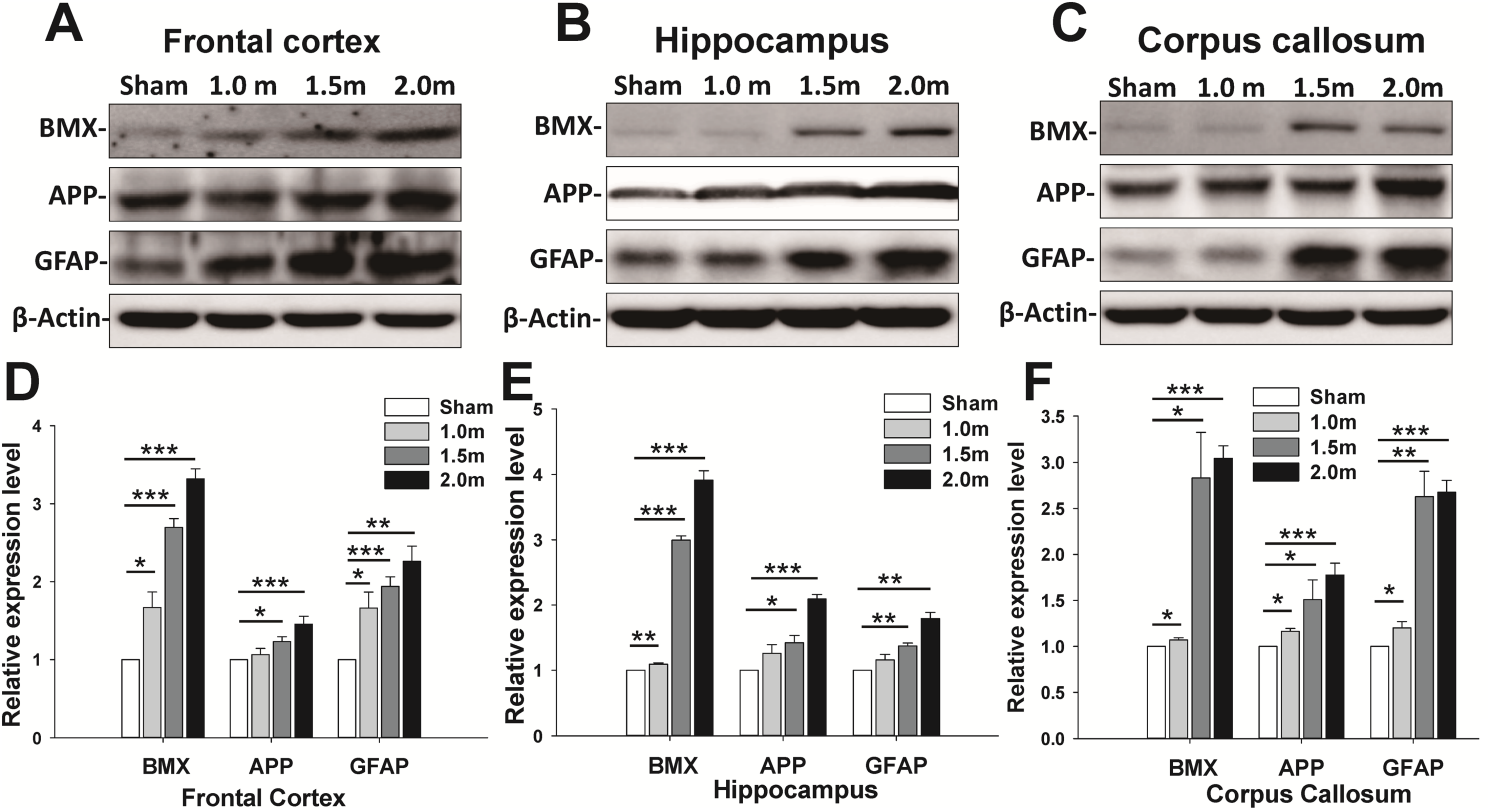
The changes in protein expression of BMX, APP, and GFAP for sham-lesion, 1-, 1.5-, and 2-m weight-drop-induced brain injury in the FC (A), H (B), and CC (C) on day 1 after injury. Western blot analysis revealed that protein expression increased in a weight-drop-height-dependent manner. Expression of BMX, APP, and GFAP in the FC (D), H (E), and CC (F) of the three weight-drop groups on day 1 post-injury. The densitometry values for protein expression as a ratio to actin were normalized to sham-lesion group. Data are represented as the mean ± SEM. *p < 0.05, **p < 0.01, ***p < 0.001 vs. sham-lesion group (*n* = 3 in each group).

**Fig 8:**
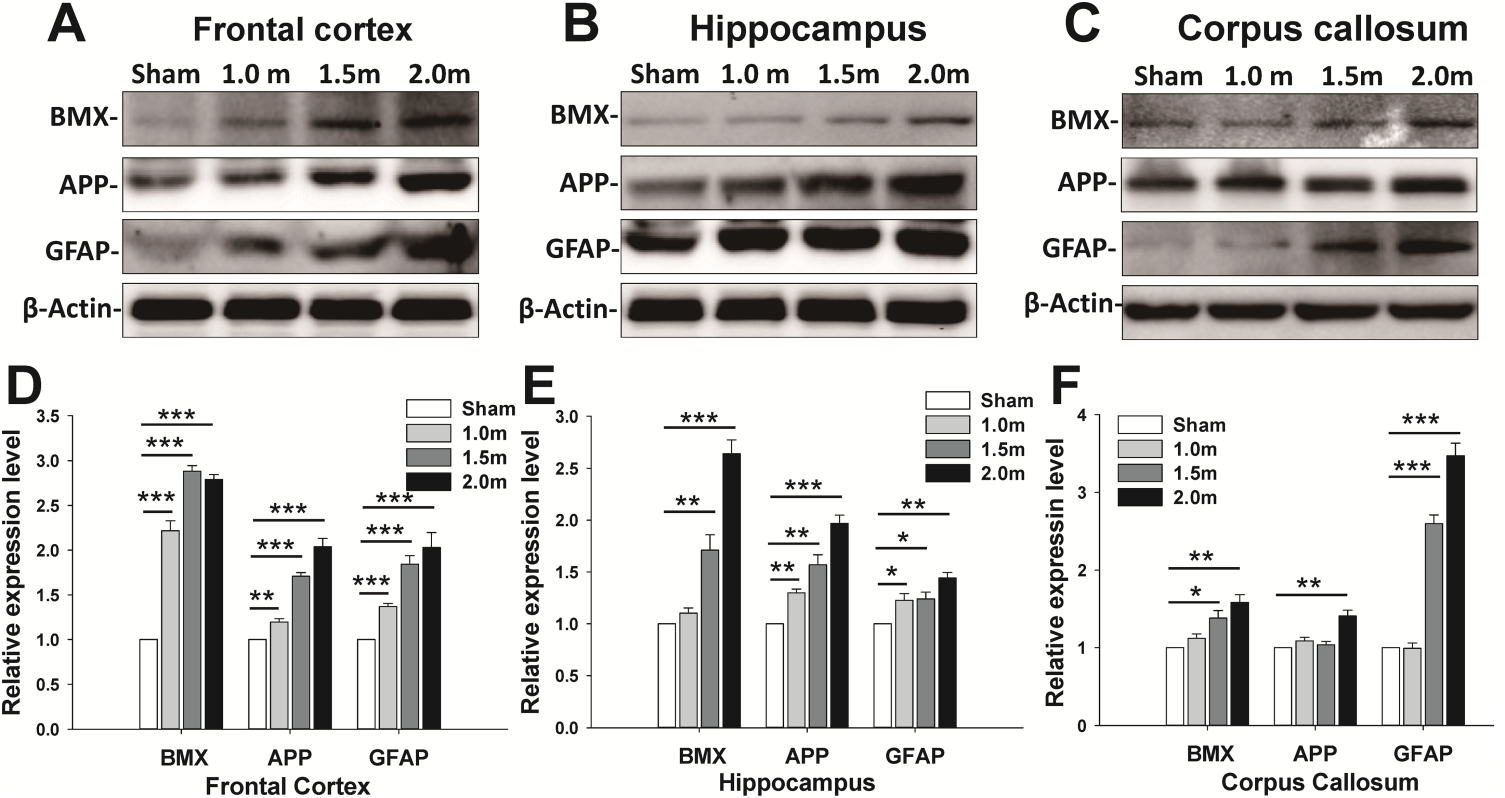
Increase in protein expression of BMX, APP, and GFAP for sham lesion, 1-, 1.5-, and 2-m weight-drop-induced brain injury in the FC (A), H (B), and CC (C) 7 days after injury. The graph shows significant upregulation of BMX, APP, and GFAP in the 1.5- and 2-m group compared with the 1-m group after weight-drop-induced brain injury. The densitometry values for protein expression as a ratio to actin were normalized to sham-lesion group. Data are represented as the mean ± SEM. *p < 0.05, **p < 0.01, ***p < 0.001 vs. sham-lesion group (*n* = 3 in each group).

**Fig 9:**
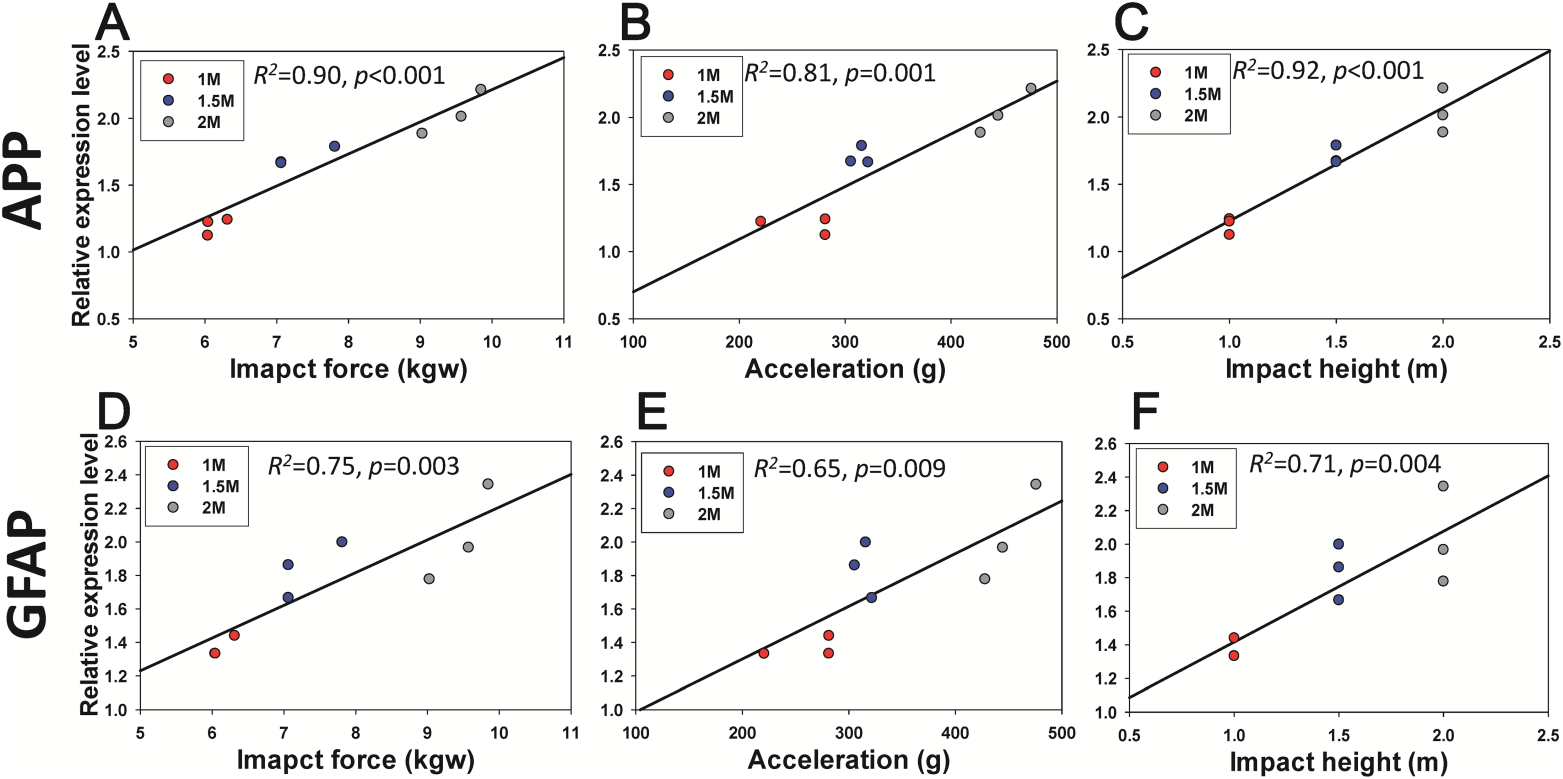
The correlation between the protein expression of frontal cortex at 7 days post-lesion and the impact force, induced acceleration and impact height during weight-drop. There was a significant positive correlation between the APP protein expression and the level of impact force (A), acceleration (B) and impact height (C). The GFAP protein expression was also found to have significant positive correlation in the level of impact force (D), acceleration (E) and impact height (F).

Because the neurological and balance impairments were observed at various impact levels, the associations of the expression of GFAP, APP, and BMX with trauma severity were examined. A Western blot analysis of the FC, H, and CC lysate revealed an increase in TBI biomarkers (BMX, GFAP, and APP) at 1 (Fig 7A–7C, S1 Fig) and 7 days (Fig 8A–8C, S2 Fig) post-injury. The protein expression of BMX, GFAP and APP increased at 1 and 7 days following impact, and the increase in the levels also differed among the three trauma severity groups. The increase in the BMX, GFAP, and APP upregulation levels upon impact showed statistically significant differences among groups with varied trauma severity at 1 (Fig 7D–7F) and 7 days (Fig 8D–8F) post-injury (*p* < 0.05, one-way ANOVA, post hoc LSD analysis). To further identify the relationship between physical impact and induced brain injury level at various weight-drop heights. We also performed the correlation tests between the inflammatory expression markers (i.e., APP and GFAP) on day 7 post-injury and impact force, peak to peak acceleration and impact height. The linear correlation analysis showed a significant positive correlation between the protein expression in APP and impact force (R^2^ = 0.90, *p* <0.001), acceleration (R^2^ = 0.81, *p* <0.001) and impact height (R^2^ = 0.92, *p* <0.001) (Fig 9A–9C). A significant positive correlation was also found between the GFAP expression level and the impact force (R^2^ = 0.75, *p* = 0.003), acceleration (R^2^ = 0.65, *p* <0.009) and impact height (R^2^ = 0.71, *p* <0.004) (Fig 9D–9F). These results indicate that higher head impact force or accelerations produced more severe brain injury.

## Discussion

In the present study, we modified Marmarou’s impact acceleration model and characterized the kinematic parameters during the weight-drop impact. The changes in impact force on the head, linear acceleration, and displacement of the rat head during impact injury of various severities were recorded and analyzed. Furthermore, we conducted a detailed experiment to identify the relationship between the impact kinematics and the changes in neurological function and motor behaviors by using the modified weight-drop TBI model. Our results demonstrate that controlling impact height and pressure can reliably induce injury of graded severity. The results also showed a highly positive correlation between the behavioral tests and recorded impact parameters. This model may serve as a translational platform for bridging human and animal studies and establishing new therapeutic strategies for TBI.

Although Marmarou’s impact acceleration model has been widely used in various TBI studies, it has been criticized for not providing highly reproducible results because of the lack of precise control over impact force and animal biomechanics and the lack of precise recording during impact [6, 18, 22]. Furthermore, the Marmarou impact acceleration model is lacking the precise control and recording during impact, which may result in a high degree of variability, making reproducibility of the injury level difficult to achieve reproduce between among different various laboratories and researchers. Thus, to eliminate the variations and standardize the protocol of this model, particularly to quantify certain aspects of TBI severity, we modified the Marmarou’s protocols and measured the weight-drop-induced impact force and acceleration or deceleration during impact. In addition, unlike the clinical setting for the classification of experimental TBI into mild, moderate, and severe levels from histological evidence and functional tests, a weight-drop protocol for inducing various TBI severity levels has less been investigated in previous studies. With the detailed functional behavior and biochemistry analysis, our results demonstrated that the severity of the induced brain injury can be predicted by the precise quantification over impact force and acceleration. Therefore, it is suggested that this protocol can reproducibly and reliably induce graded severity of brain injury as well as graded neurological and motor impairments. Furthermore, where the method and instrument developed in the present study is available, it can be applied to gain the knowledge and offer further insights into understanding the relationship between the pathophysiologic changes following TBI and head mechanical response (e.g., force and acceleration) during impact.

When a suitable diseased animal model is used, understanding the neurological outcomes after injury is crucial. We employed a time-course analysis of behavioral and biochemical recordings, which highly correlated with the impact force and the kinematic results during impact. We selectively used three traditional fall heights, which were obtained from conditions identical to those in the original impact acceleration model by Marmarou [4, 5]. The induction of behavioral and neuropathological changes in our study is similar to that of earlier studies that used identical or similar models [17, 18, 23]. We observed that the neurological scores and functional beam balance varied with impact force and acceleration; an impact height of less than 1 m produced no or mild impaired functional behaviors, whereas increasing the impact height to 1.5 and 2.0 m significantly increased impaired functional behaviors. Although the induced severity of the current TBI weight-drop model can be determined by the mNSS evaluation conducted 1 day post-lesion, no universal neurological examination system for the brief identification of severity, such as the Glasgow Coma Scale in patients with TBI, has been widely adopted for rats. Therefore, depending on current setting in this study, the mechanical injury parameters such as impact force and induced head acceleration changes in combination with other histological or biochemical evidence and functional tests could provide the most reliable measures for classifying an experimental TBI model into mild, moderate, or severe levels.

Functional behavior analysis is crucial in TBI research and we demonstrated that the modified weight-drop model may induce the severity-dependent neurological and motor behavioral disturbances. The multiple behavioral and biochemical testing during the course of trauma progression are warranted to reveal the levels of brain injury and motor performance, as well as to determine whether the weight-drop model induced functional deficits are stable or spontaneously recover over time. Whereas previous research has examined the behavioral alterations arising from various falling height of weight-drop method, the time-course changes of neurological and balance function of varying levels of brain injury remained not fully characterized. Thus, we applied general neurological and beam balance test following weight-drop at different time points to observe the changes of functional outcome. One day after TBI lesion, except for the 1-m fall height group, 1.5 and 2-m groups caused temporary motor balance deficits. Over 7 days, although there were spontaneous recoveries for the three fall height groups following TBI lesion, the neurological and balance function still did not reach the pre-surgery level in the 2-m weight-drop group. This implies that following severe weight-drop induced TBI, the neurological and balance function were profoundly altered. The neurological and motor deficits produced by our modified weight-drop methods are consistent with the previous reports demonstrating significant motor impairments following lateral fluid percussion and controlled cortical impact brain injury model [24–28]. In addition to the motor disturbance, although we did not measure the cognitive function after weight-drop lesion, previous studies have reported that the severity of cognitive deficits is related to the impact acceleration injury level [28]. Based on our biochemistry analysis, the neuronal damage throughout the hippocampus was present after weight-drop lesion at two time points examined, suggesting that the impaired cognitive function in rats after weight-drop could be associated with neuropathological changes in the hippocampus.

Animal models are essential for studying the biomechanical, cellular, and molecular aspects of human TBI that cannot be addressed in a clinical setting and for developing and characterizing novel therapeutic interventions. We used three markers (BMX, APP, and GFAP) to represent inflammation and axon injury following TBI. Our results support the association of BMX, APP, and GFAP upregulation with trauma severity in rats. Following weight-drop-induced brain injury, an increase in the expression of these proteins in the injured brain area was founded. The association between the BMX, APP, and GFAP expression levels and severity of injury was demonstrated using various fall heights. We found that the amount of neural inflammation and axonal injury varied with impact force and acceleration; a low impact height (1 m) produced little or no axonal injuries and a higher impact height (1.5–2 m) significantly increased these markers of inflammation and axonal injury. Notably, the changes in the levels of these markers were apparent early at day 1 and remained elevated for at least 1 week post TBI-lesion, indicating that the inflammation and axonal injuries appeared in acute or subacute stages of TBI. Furthermore, with the spontaneous recovery, the level of balance function was not significant in the 1 and 2 m weight-drop groups. However, the biochemistry biomarkers appear to be still sensitive detection methods when compared with the behavioral tests, indicating that the subsequent increases in the levels of TBI biomarkers on day 7 post-lesion also validate the brain injury level. These results suggest that neuroinflammation or axonal damage following weight-drop-induced TBI may become detectable at acute, subacute and chronic time points following injury and may develop in a progressive manner.

Consistent with earlier studies, the increased APP, GFAP and BMX metabolites are observed following brain injury using similar Marmarou acceleration weight drop model [17, 18, 29, 30] or other experimental TBI models, such as controlled cortical impact [12] or fluid percussion [24, 27] models of brain injury. However, when compared with other TBI models, the changes of TBI markers showed different pattern in the injury site. For example, when performing 1.5 atm fluid percussion injury in rats, it does not cause visible neuronal loss in the hippocampus and neocortex, but gives rise to a robust inflammatory response (as indicated by enhanced GFAP and Iba1 immunoreactivity) in the corpus callosum and the thalamus [24]. For the characterization of time-course changes in TBI markers after brain injury, an earlier study found that the APP immunoreactivity peaked at 1 day, declined at 3day in the cortex, subcortical white matter, external capsule and the hippocampus after central fluid percussion injury in mice. The GFAP reactivity was also observed in cortical regions peaking at 3 days post-injury [27]. Moreover, for the controlled cortical impact injury rat model, a dramatic increase in APP immunoreactivity in the hippocampus and cortex was found at 1 day after lesion and sustained up to 3 days post-injury [31]. The increase in BMX expression level was observed as early as 3 hours and maintained for 3 days or more. In contrast, the level of expression for GFAP increased nearly at 4 days post-trauma [12]. Such discrepancy between studies could be due to the different types of TBI animal model, different severity of the brain injury induction, and the variability of protocols. Although direct comparisons between the earlier published and present results during the course of trauma progression must be made with caution, the expression of such markers may play important roles in the pathogenesis of TBI to represent the levels of inflammatory or axonal injury and thus act as indicators of trauma severity.

The present study provides such evidence and shows a significant correlation between the neurologic dysfunction severity and the mechanical impact magnitude. It is noted that the severity of neurological function, beam balance and brain damage at 1 day or 7 days post-lesion have been shown to strongly correlate with the increased impact force, acceleration and fall height during impact, as determined by several specific sensors. Furthermore, the impact force and peak to peak acceleration showed better correlation with neurobehavioral function than impact height. Although initial impact height were consistent during impact, this result indicates that traditional weight-drop protocol for inducing graded injury severity by adjusting fall height could still have high variability. It is suggested that the graded severity of brain injury can be predicted by the real time measurement of impact force and acceleration. Otherwise, our results demonstrate that stringent control over impact force and acceleration can reproducibly and reliably induce graded severity of brain injury.

## Conclusions

In the present study, we developed a characteristic analysis method for detecting kinematic data for various severities of TBI in the weight-drop-induced TBI model. By using this model, we showed that various graded impact forces and accelerations can produce graded neurological and motor impairments as well as axonal inflammation and injury. This method entails using newly developed quantitative measures to reduce the variation and increase the reproducibility of the weight-drop-induced TBI model. This method would be useful for studying the pathophysiology of TBI and developing therapeutic strategies for TBI.

## Supporting information

S1 Data The measured value of impact force, acceleration, deceleration and peak to peak acceleration during weight-drop under various impact height. (PDF)

S1 Fig Full blot images for Western blot presented in Fig 7. (PDF)

S2 Fig Whole blot images of Western blots presented in Fig 8. (PDF)

S1 Video Supplementary video of weight drop induced traumatic brain injury model. The demonstrated video was recorded at 300 frames/s during weight-drop experiment. The acceleration, impact force, and displacement during the impact were measured using an accelerometer, a pressure sensor, and a high-speed camera, respectively.
(WMV)
