# Correction: Relationship of mechanical impact magnitude to neurologic dysfunction severity in a rat traumatic brain injury model

**DOI:** 10.1371/journal.pone.0182300

**Published:** 2017-07-24

**Authors:** Tsung-Hsun Hsieh, Jing-Wei Kang, Jing-Huei Lai, Ying-Zu Huang, Alexander Rotenberg, Kai-Yun Chen, Jia-Yi Wang, Shu-Yen Chan, Shih-Ching Chen, Yung-Hsiao Chiang, Chih-Wei Peng

Fig 1 and Fig 2 are incorrectly swapped. Please see the correct Figs 1 and 2 and their captions here.

**Fig 1:**
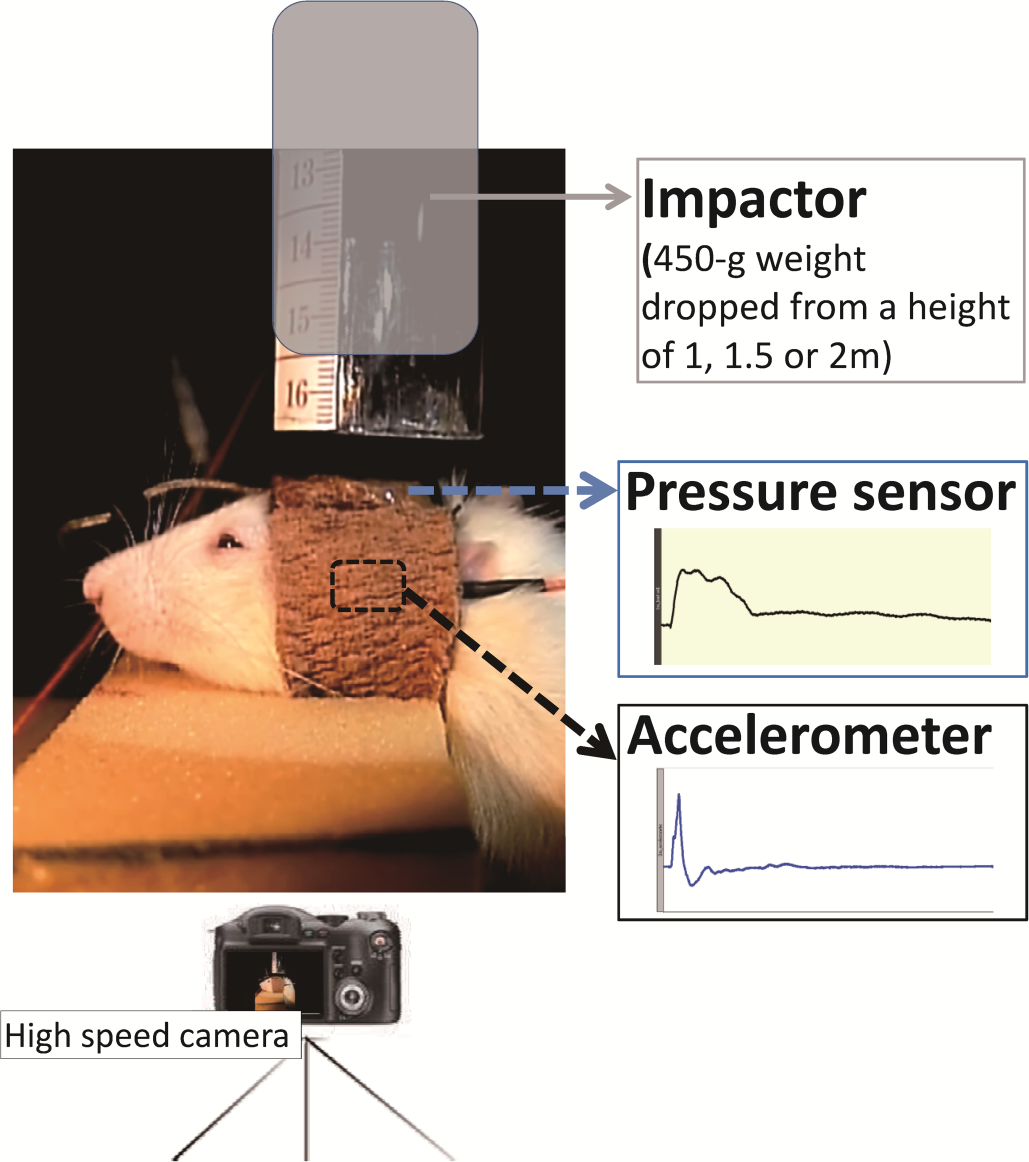
Instrumentation setup of the modified weight-drop-induced head injury model. Kinematic information during impact was captured using a high-speed camera at a rate of 1200 frames/s. The impact force was measured using the miniature load cell that was fixed to the central portion of the skull vault of the rat. The linear acceleration response of the rat head was recorded using a modified accelerometer.

**Fig 2:**
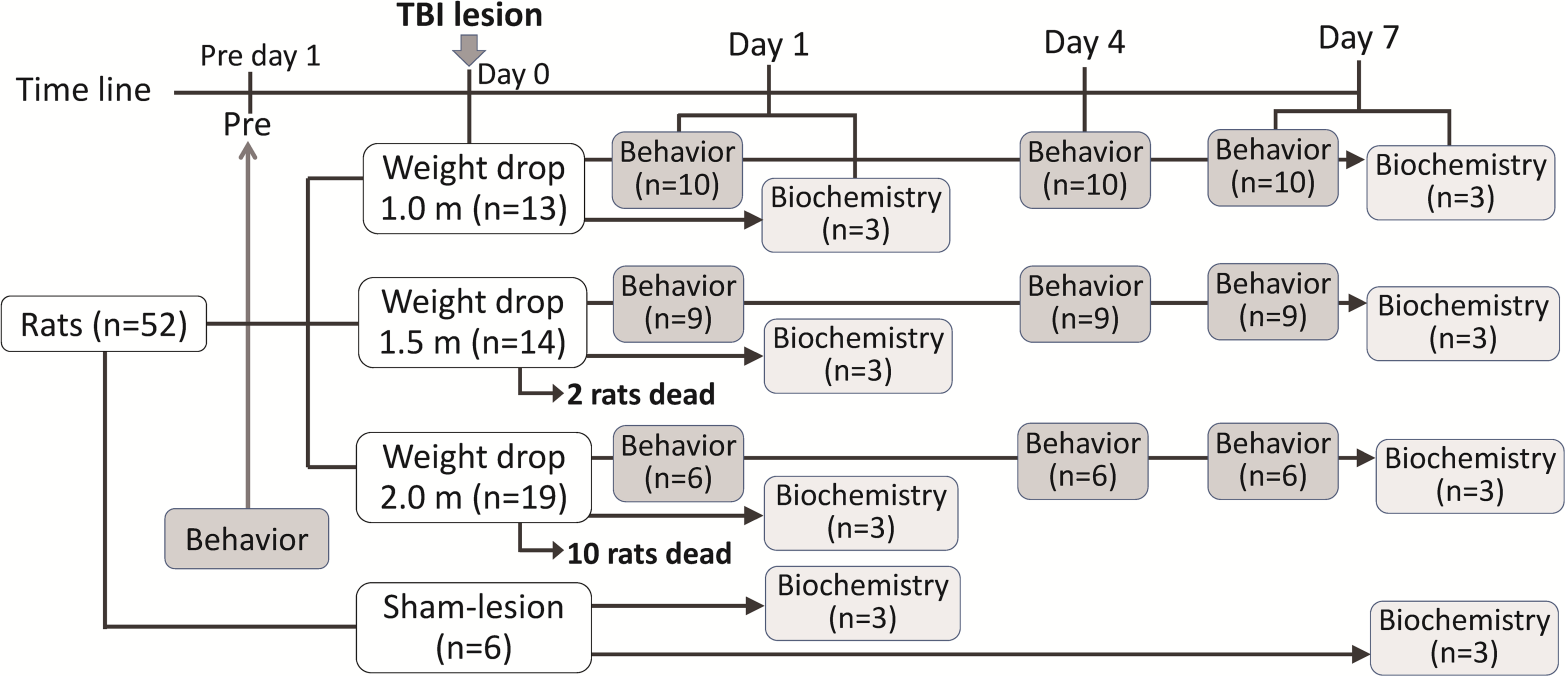
Study design of time-course analysis of behavioral and biochemical recordings following TBI lesion. Behavioral tests including mNSS and beam walking tests were performed before lesion and on days 1, 4, and 7 post-lesion. Western blotting tests were performed on days 1 and 7 post-lesion to quantify the injury severity following weight-drop-induced head injury.
