# Supplementary material for: Relationship of mechanical impact magnitude to neurologic dysfunction severity in a rat traumatic brain injury model
Source: PLoS One. 2017 May 26;12(5):e0178186. doi: 10.1371/journal.pone.0178186 (PMC5446124; doi:10.1371/journal.pone.0178186)
Supplement: S1 Data — (PDF) [file pone.0178186.s001.pdf]

Impact Height (m)	Measured impact force (kgw)		Measured Acceleration (g)	Measured Deceleration (g)	Peak to Peak Acceleration (g)
1.000	6.312		237.210	73.660	310.869
1.000	6.038		220.036	62.479	282.515
1.000	6.043		196.720	33.530	230.250
1.000	4.960		222.909	58.297	281.206
1.000	7.250		213.690	67.460	281.150
1.000	4.770		172.782	36.229	209.011
1.000	6.549		189.313	31.086	220.399
1.000	6.833		234.827	55.602	290.429
1.000	6.708		196.790	61.080	257.870
1.000	6.615		175.296	48.860	224.156
1.500	7.061		194.674	48.936	243.609
1.500	7.810		288.464	66.815	355.279
1.500	7.061		264.262	58.393	322.655
1.500	6.358		249.361	56.042	305.403
1.500	7.352		284.422	37.208	321.630
1.500	8.640		246.851	68.858	315.709
1.500	6.980		186.940	38.991	225.931
1.500	7.930		299.039	70.759	369.798
1.500	6.289		207.450	49.273	256.723
2.000	8.520		346.137	61.466	407.603
2.000	9.573		378.849	96.963	475.812
2.000	9.847		338.424	89.334	427.758
2.000	9.028		383.163	57.008	440.170
2.000	10.417		404.176	101.926	506.102
2.000	9.170		370.914	73.526	444.441

Impact Height	Averaged Impact force	S.E (Impact force)	Impact Height	Averaged Acceleration	S.E (Acceleration)
1.000	6.208	0.252	1.000	205.957	7.335
1.500	7.276	0.252	1.500	246.829	13.964
2.000	9.426	0.272	2.000	370.277	9.977

Impact Height	Averaged Deceleration	S.E (Deceleration)
1.000	52.828	4.694
1.500	55.030	4.142
2.000	80.037	7.680

Impact Height	Averaged peak to peak acceleration	S.E (peak to peak acceleration)
1.000	258.785	11.178
1.500	301.860	16.538
2.000	450.314	14.414
