# Supplementary figures and images for: Relationship of mechanical impact magnitude to neurologic dysfunction severity in a rat traumatic brain injury model
Source: PLoS One. 2017 May 26;12(5):e0178186. doi: 10.1371/journal.pone.0178186 (PMC5446124; doi:10.1371/journal.pone.0178186)

# Frontal Cortex

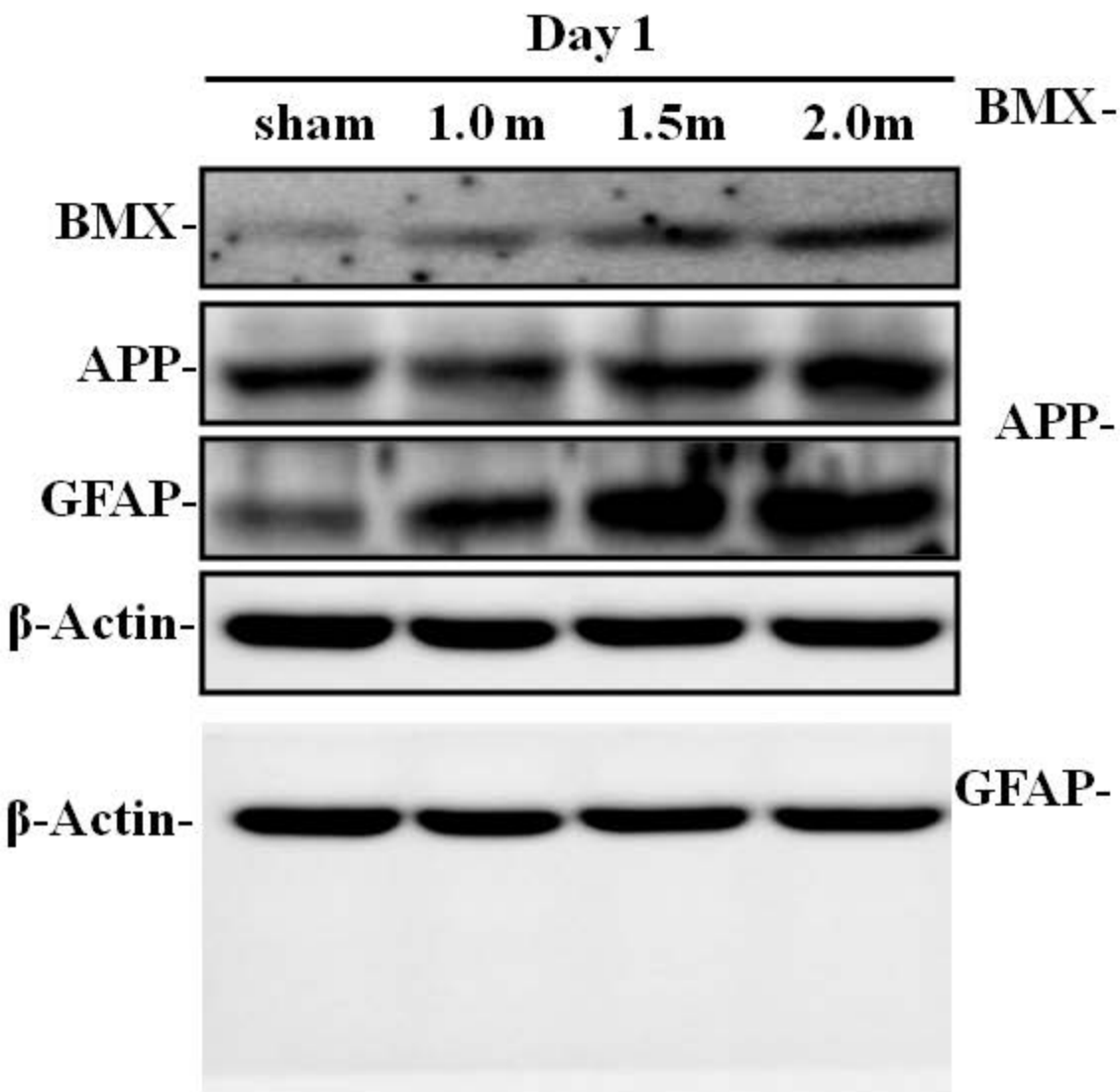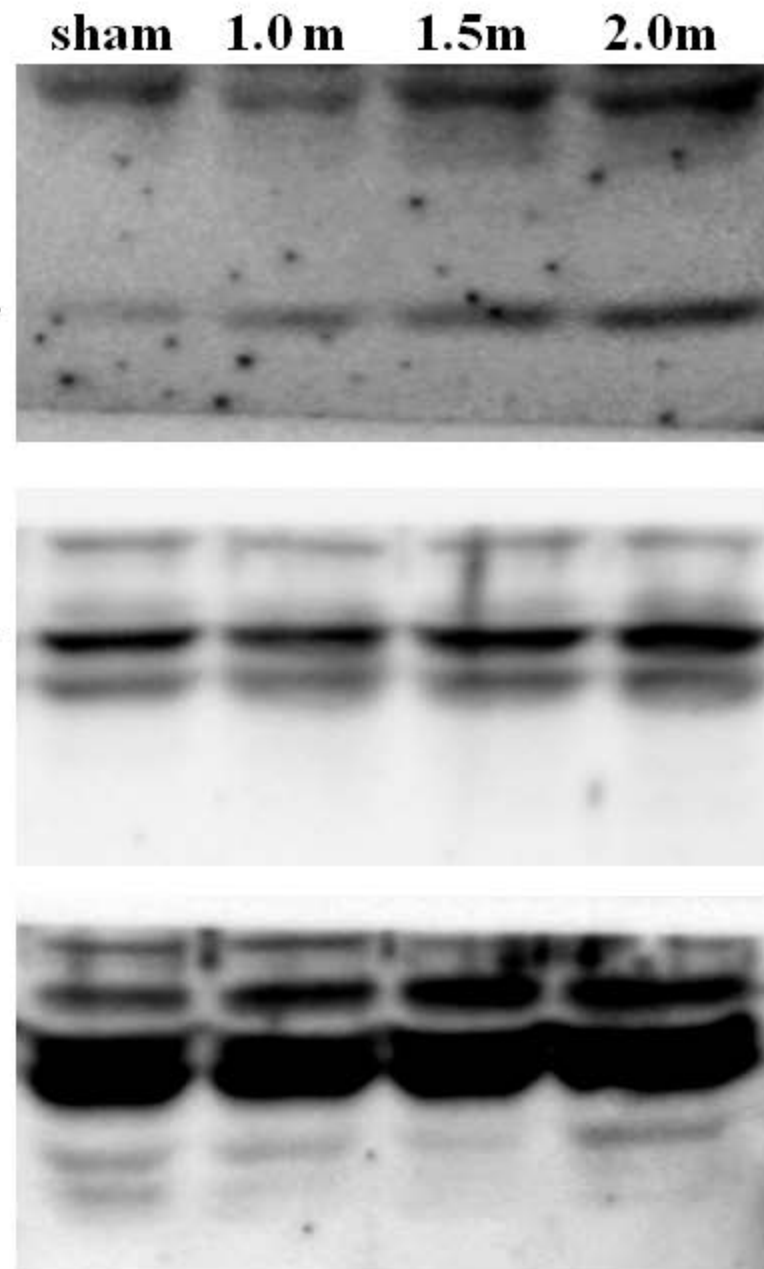

# Hippocampus

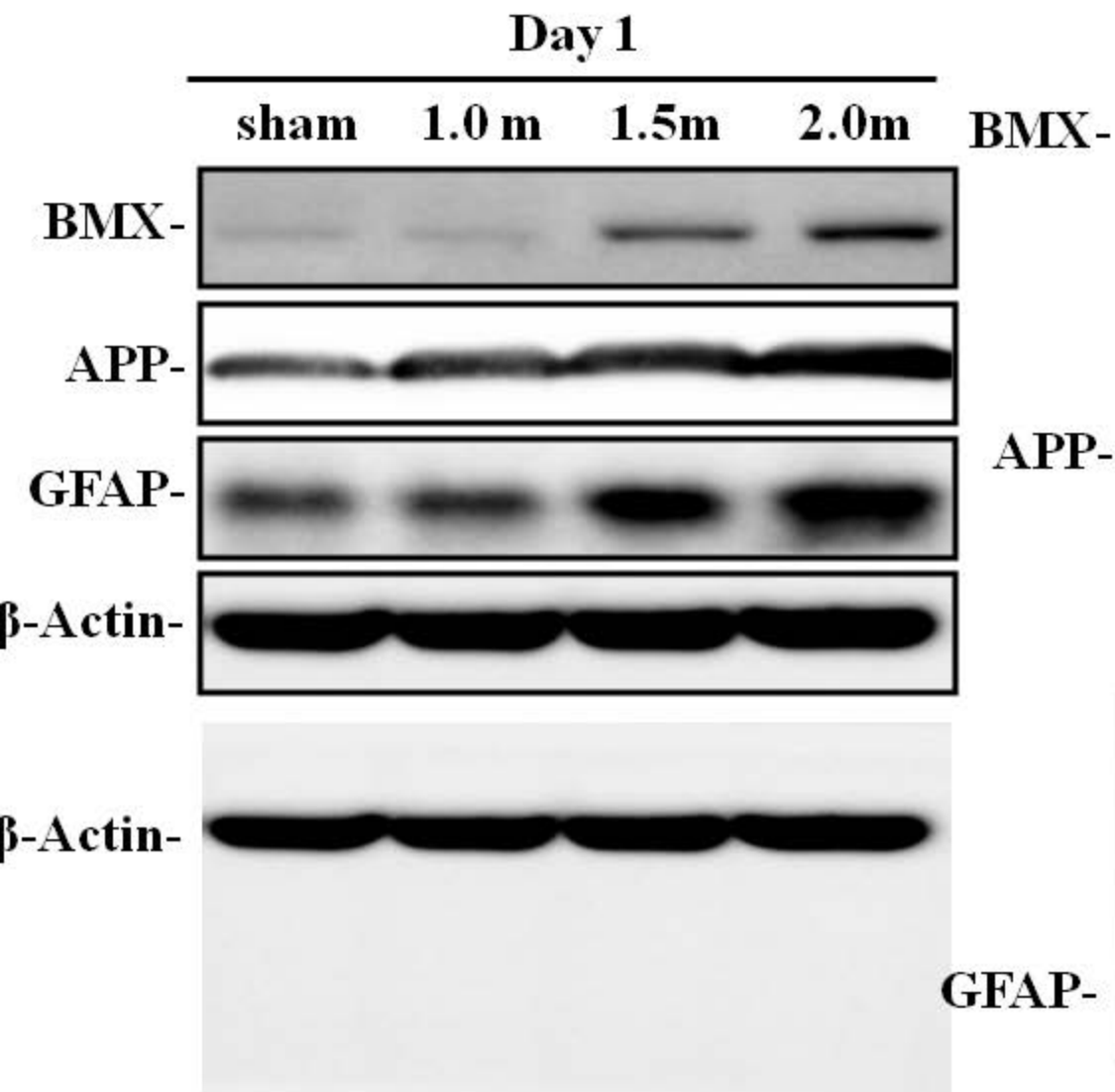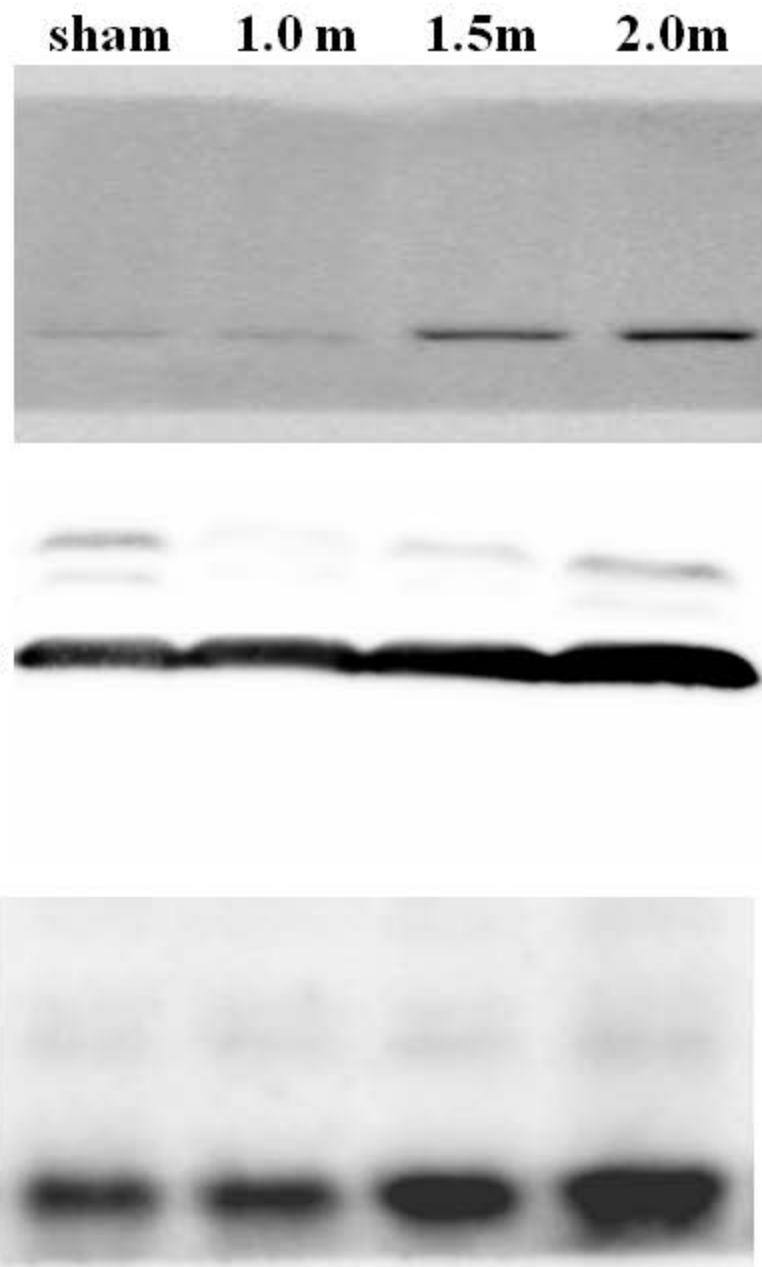

# Corpus Callosum

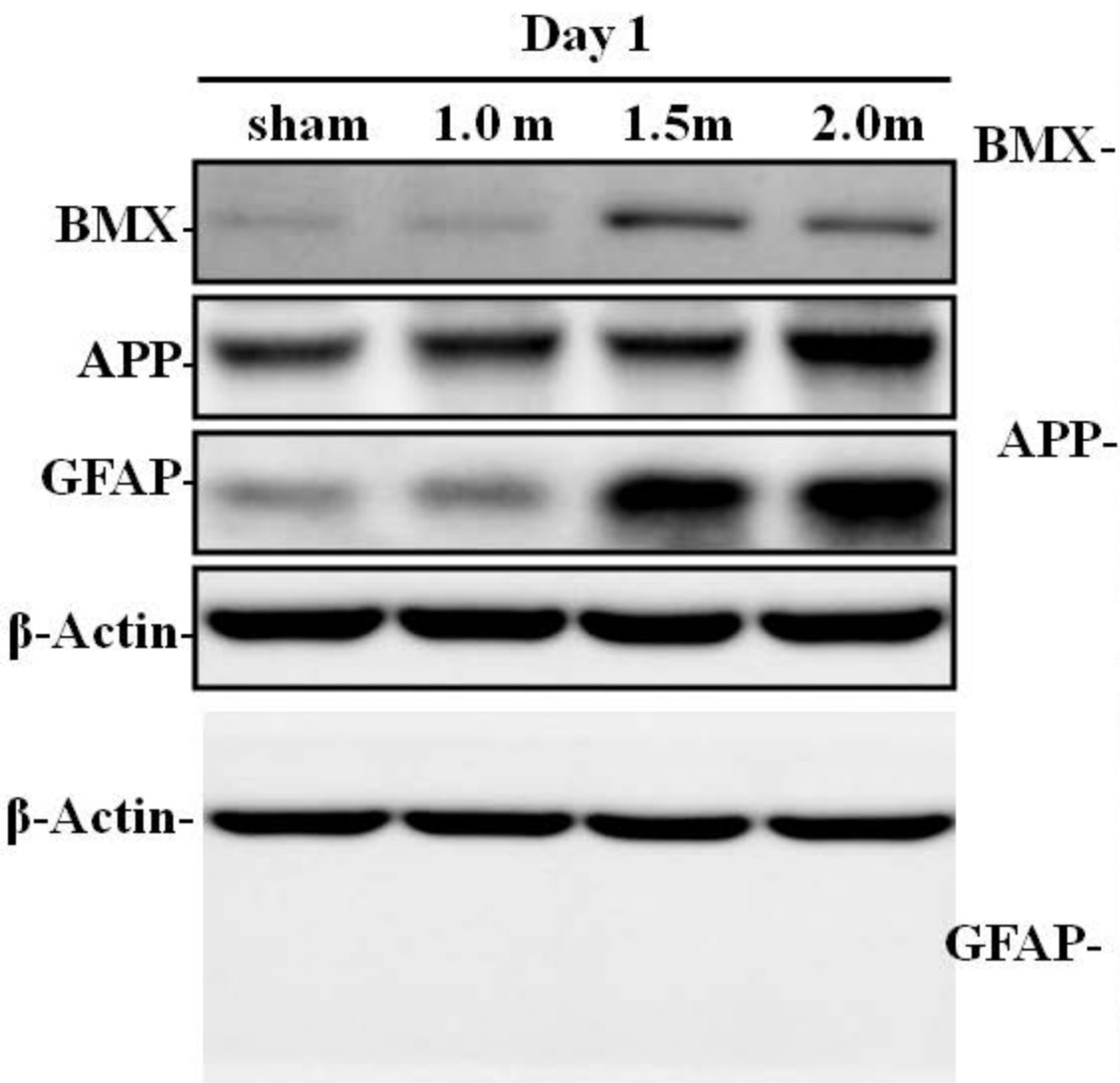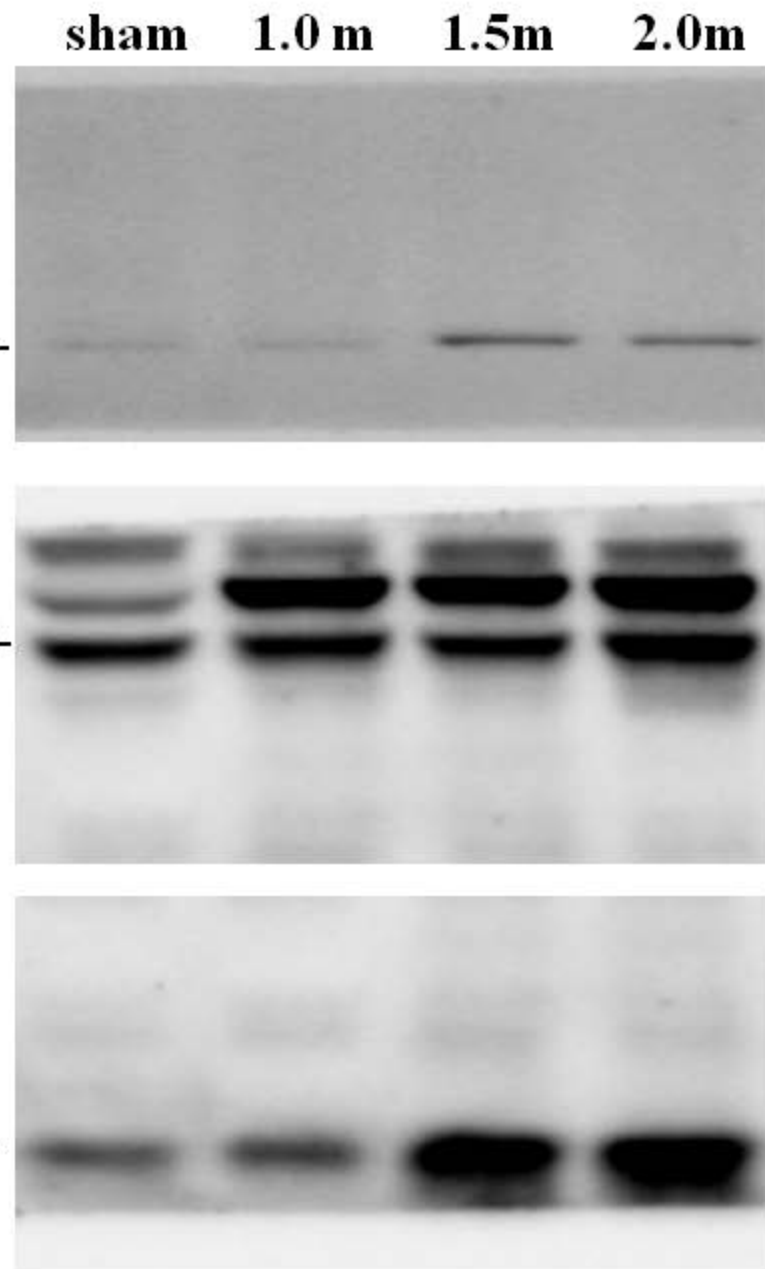

Supplement: S1 Fig — (PDF) [file pone.0178186.s002.pdf]

# Frontal Cortex

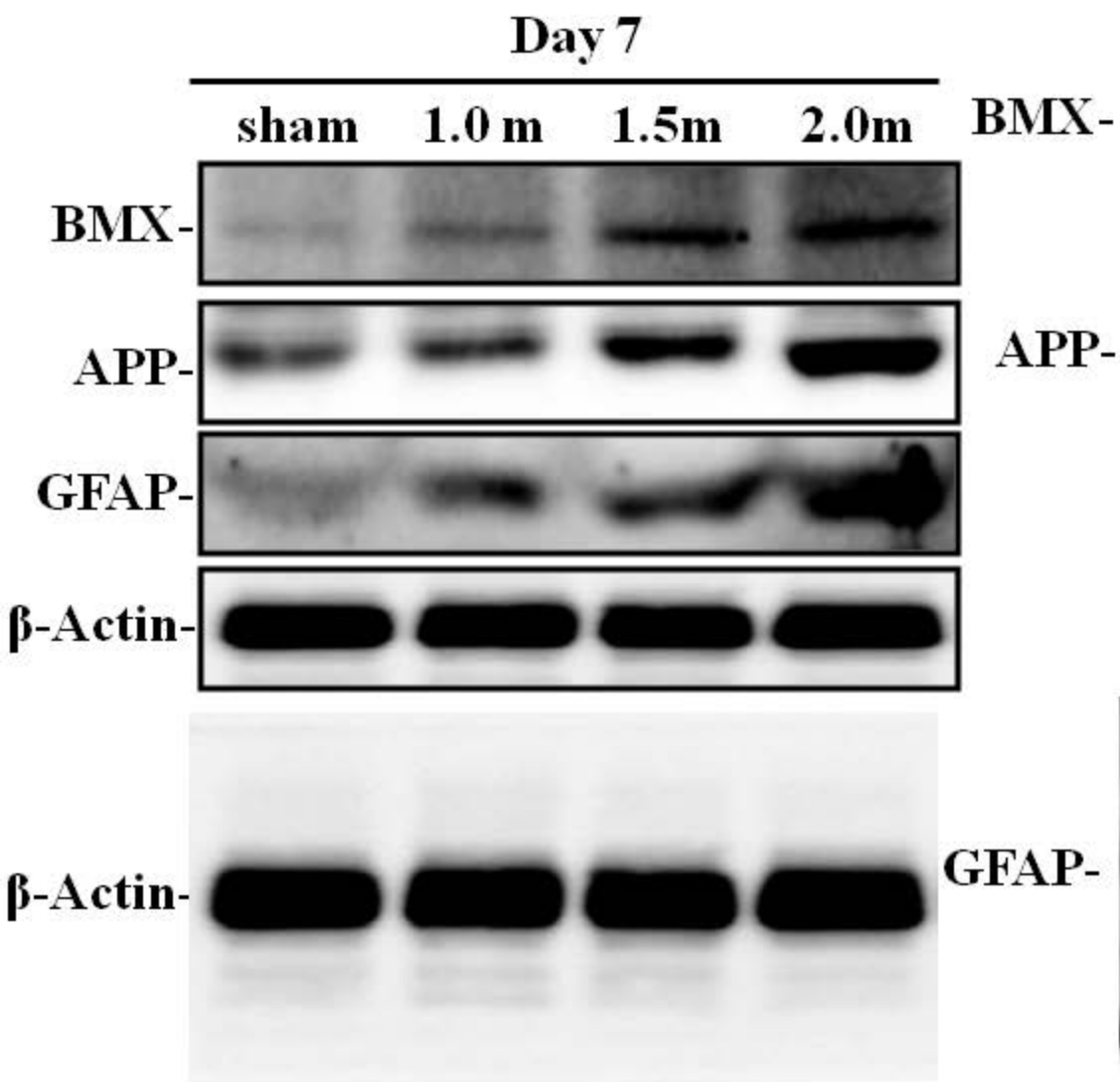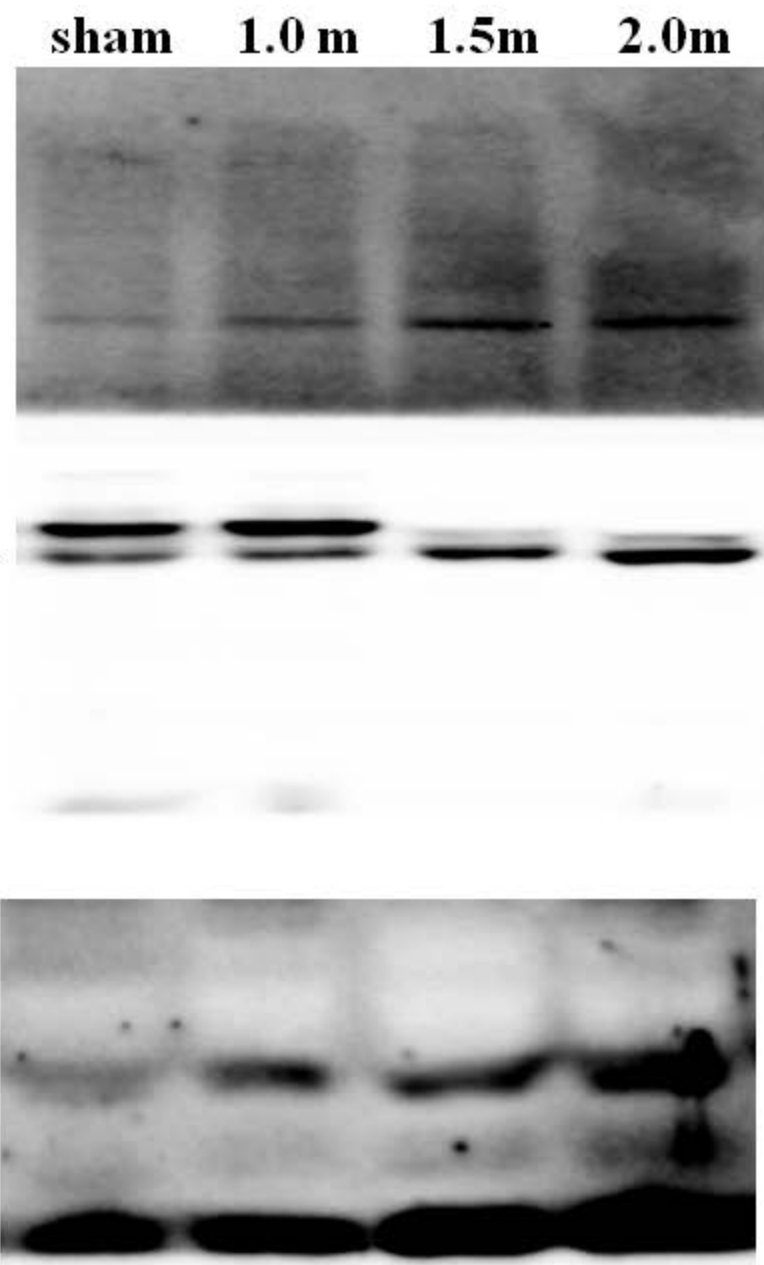

# Hippocampus

Day 7

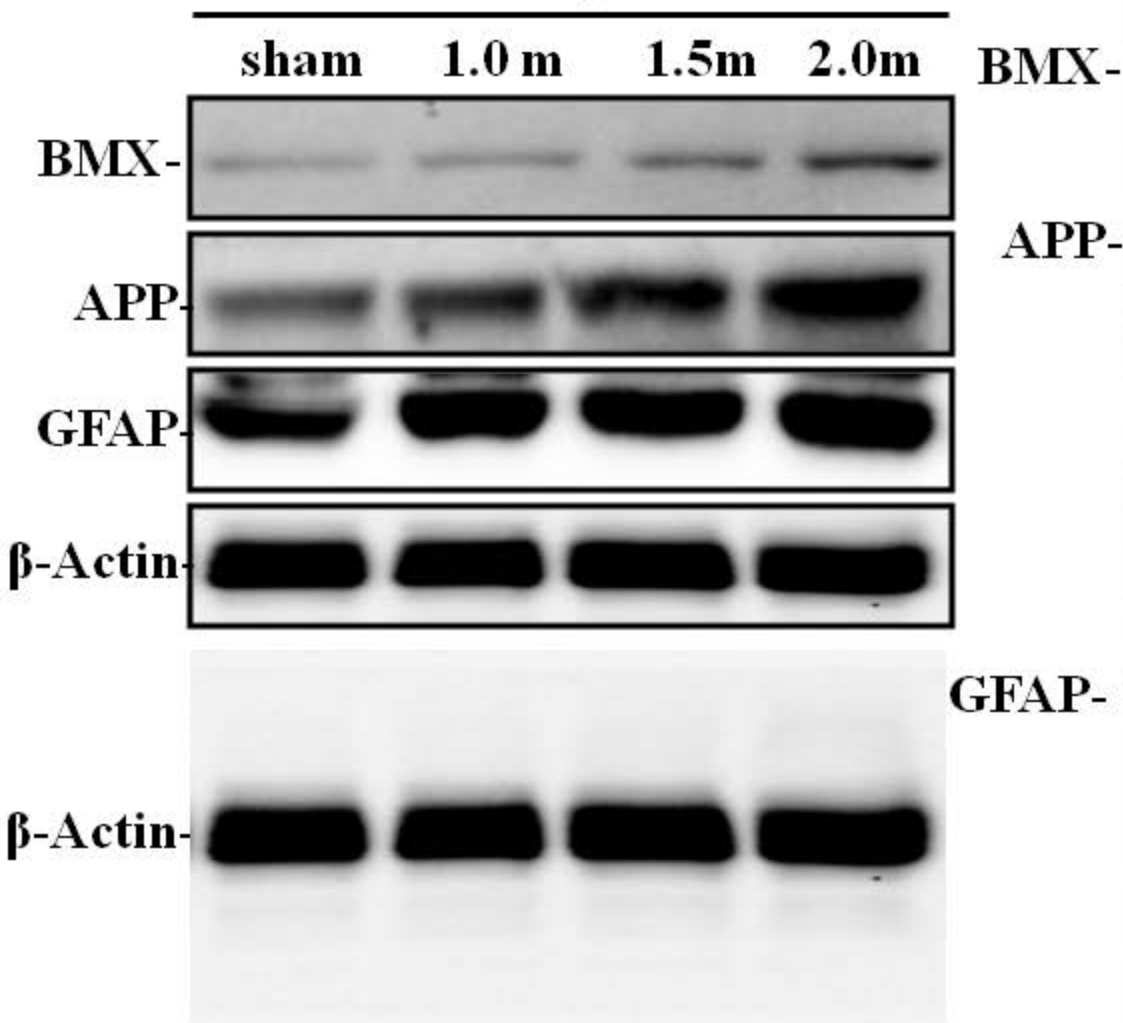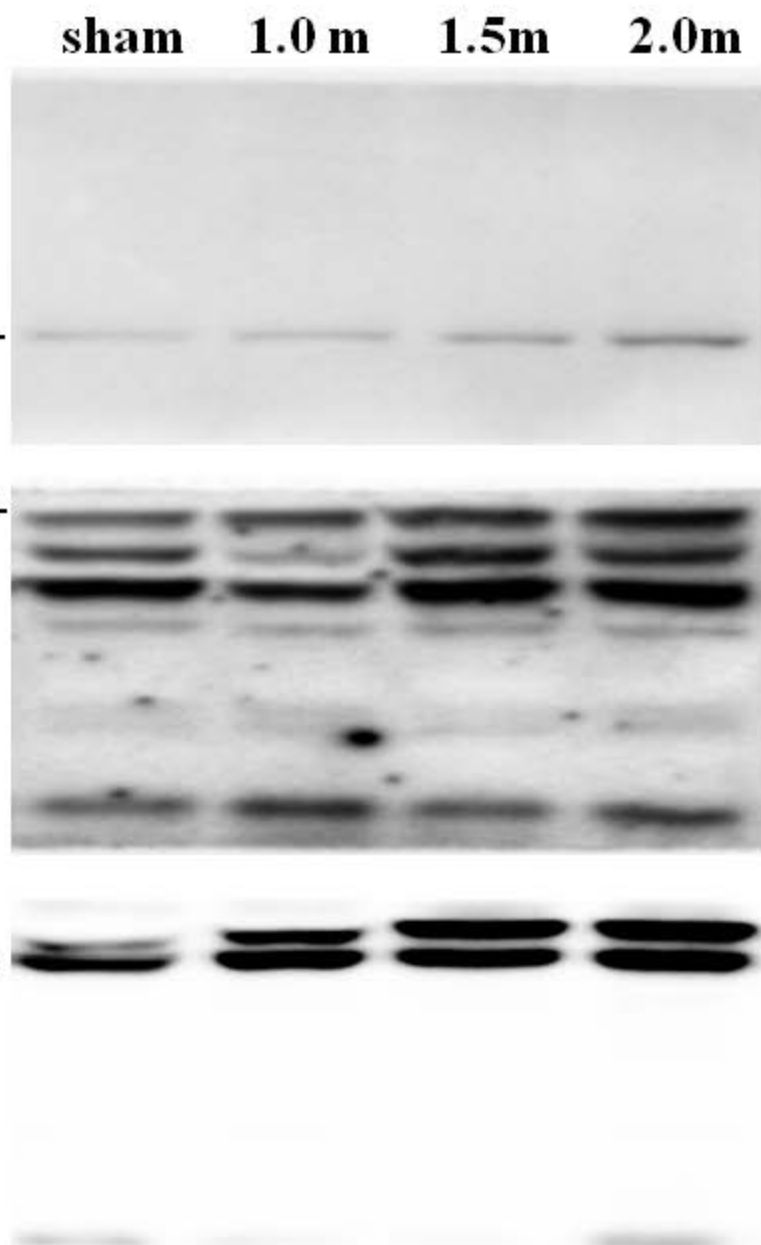

# Corpus Callosum

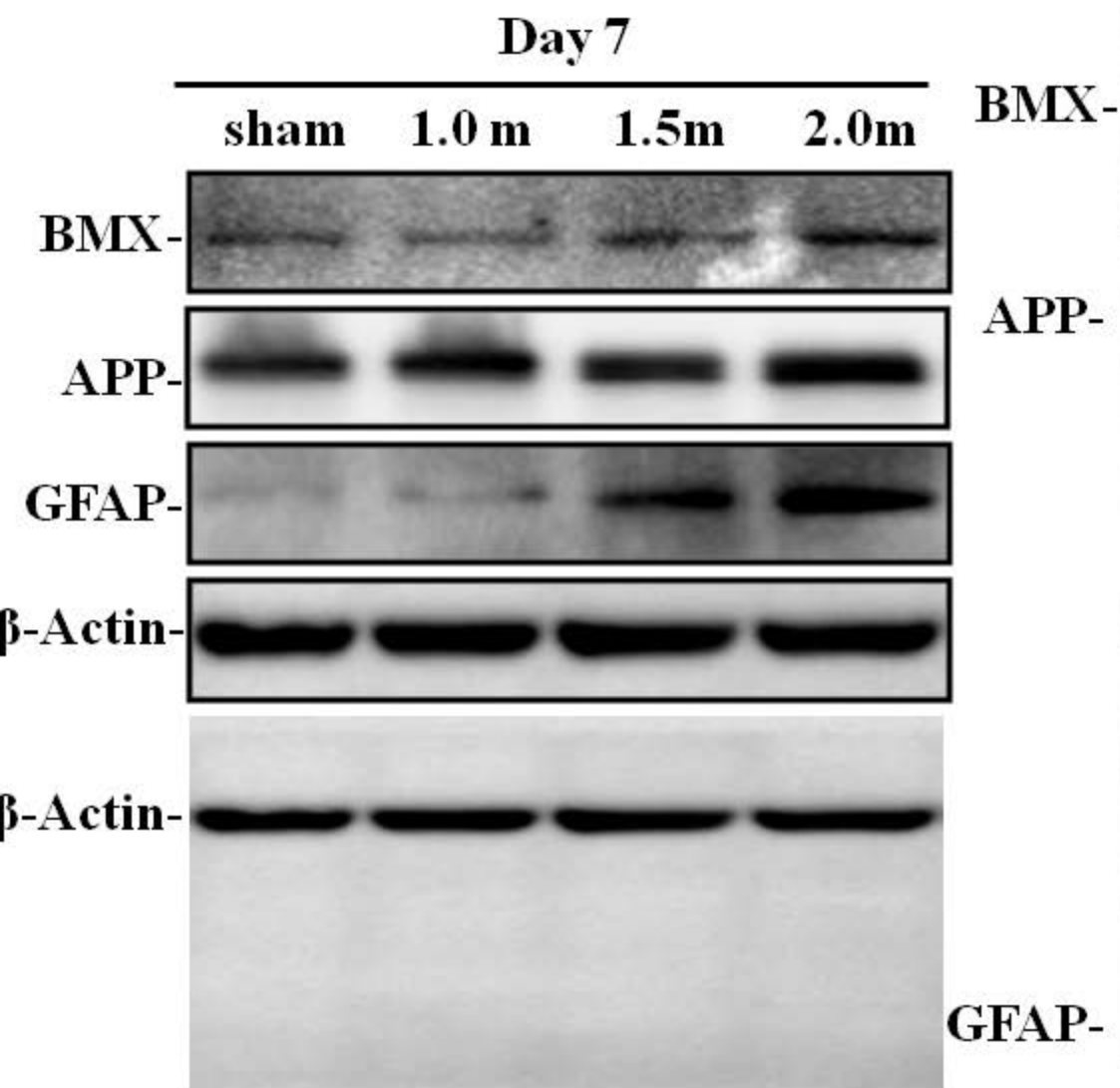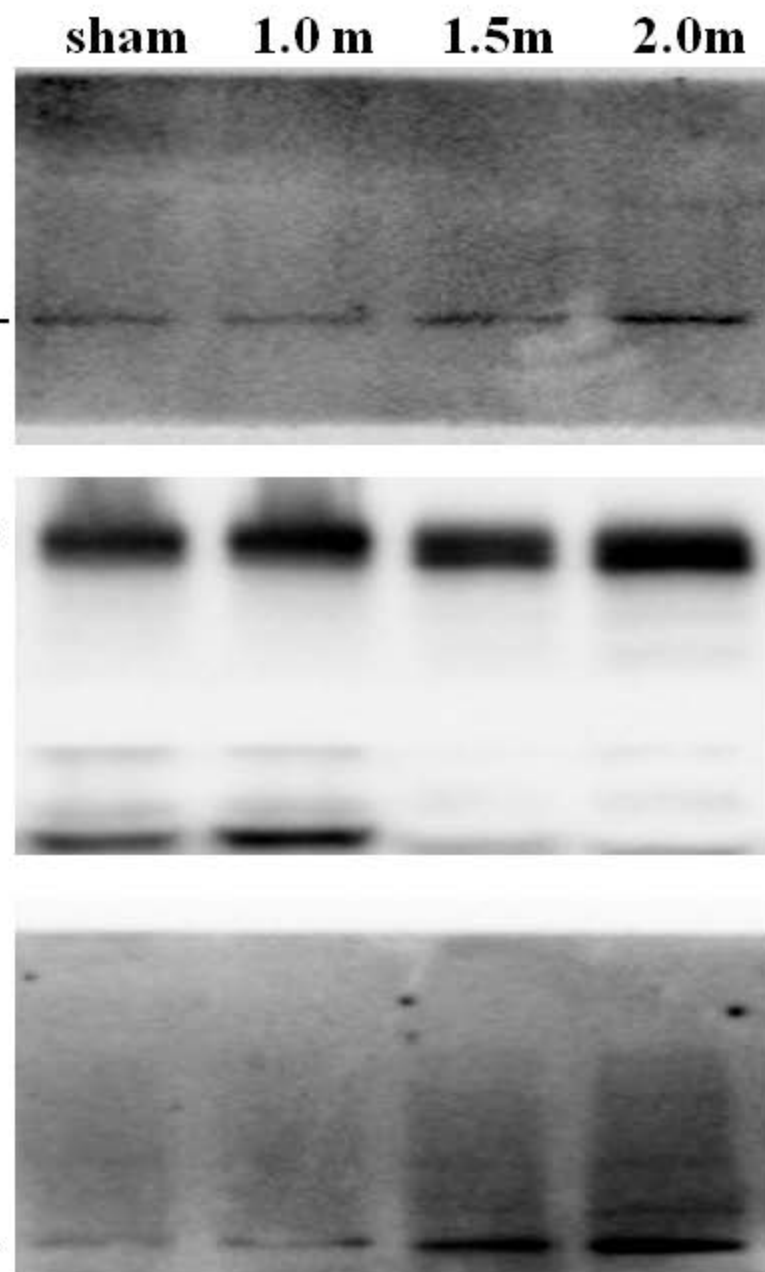

Supplement: S2 Fig — (PDF) [file pone.0178186.s003.pdf]
